# Biotic and abiotic factors distinctly drive contrasting biogeographic patterns between phyllosphere and soil resistomes in natural ecosystems

**DOI:** 10.1038/s43705-021-00012-4

**Published:** 2021-04-14

**Authors:** Zhen-Zhen Yan, Qing-Lin Chen, Chao-Yu Li, Bao-Anh Thi Nguyen, Yong-Guan Zhu, Ji-Zheng He, Hang-Wei Hu

**Affiliations:** 1 School of Agriculture and Food, Faculty of Veterinary and Agricultural Sciences, The University of Melbourne, Parkville, VIC Australia; 2 Key Laboratory of Urban Environment and Health, Institute of Urban Environment, Chinese Academy of Sciences, Xiamen, China

**Keywords:** Environmental sciences, Environmental microbiology

## Abstract

The phyllosphere and soil are two of the most important reservoirs of antibiotic resistance genes (ARGs) in terrestrial ecosystems. However, comparative studies on the biogeographic patterns of ARGs in these two habitats are lacking. Based on the construction of ARG abundance atlas across a > 4,000 km transect in eastern and northern Australia, we found contrasting biogeographic patterns of the phyllosphere and soil resistomes, which showed their distinct responses to the biotic and abiotic stresses. The similarity of ARG compositions in soil, but not in the phyllosphere, exhibited significant distance-decay patterns. ARG abundance in the phyllosphere was mainly correlated with the compositions of co-occurring bacterial, fungal and protistan communities, indicating that biotic stresses were the main drivers shaping the phyllosphere resistome. Soil ARG abundance was mainly associated with abiotic factors including mean annual temperature and precipitation as well as soil total carbon and nitrogen. Our findings demonstrated the distinct roles of biotic and abiotic factors in shaping resistomes in different environmental habitats. These findings constitute a major advance in our understanding of the current environmental resistomes and contribute to better predictions of the evolution of environmental ARGs by highlighting the importance of habitat difference in shaping environmental resistomes.

## Introduction

The links between microbial distribution patterns and their underlying drivers are a core topic of microbial ecology.^1^ Previous studies have revealed that the distribution patterns of soil microbial communities can vary markedly across space.^2–6^ Endemic abiotic factors, including climate,^4,6^ the availability of growth-limiting nutrients,^2,3^ soil salinity,^7^ and soil pH,^5^ have been demonstrated to be important in determining the biogeographic patterns of soil microbial communities. In addition to abiotic factors, the inter-kingdom interactions among co-occurring microbial communities also play important roles in governing the biogeographic patterns and functional diversity of soil microbial communities.^8–10^ For example, the antagonism between bacterial and fungal communities can lead to the production of antibiotics by some bacterial and fungal cells, which exert selection pressure for the evolution of antibiotic resistance genes (ARGs).^8,11^ ARGs, which are estimated to have existed in natural ecosystems since 2 Gyr ago, play important roles in ecosystem multifunctionality (i.e. multiple ecosystem functions and services).^12–14^ Other than as a defense strategy of bacteria to thwart the onslaught of antibiotics,^15^ ARGs are associated with a variety of other ecosystem functions such as trafficking signals, facilitating the detoxification of metabolic intermediates and enhancing bacterial pathogenicity.^14,16^ Therefore, an adequate evaluation of the biogeographic patterns of the resistomes (the collection of ARGs in communities of both pathogenic and non-pathogenic bacteria^17^) and their main driving factors is critical to advance our understanding of the spread and evolution of antibiotic resistance in natural settings under the on-going global change.

**Fig. 1:**
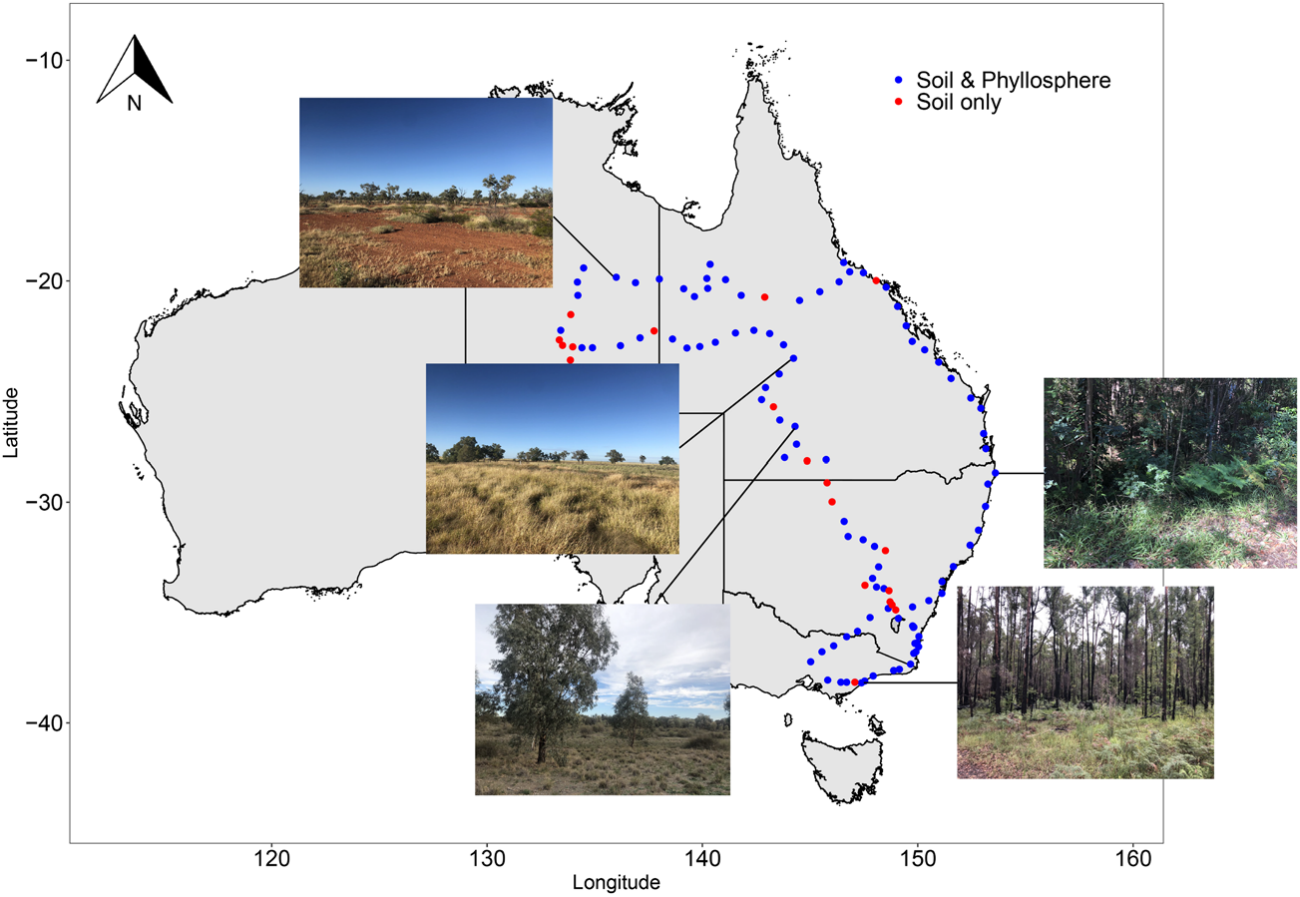
Locations and photos of the sampling sites. The blue spots represent the 100 sites where both plant and soil samples were collected. The red spots represent the 19 sites where only soil samples were collected.

The phyllosphere (the aerial part of plants)^18^ and soil^19,20^ have been ranked as two of the most important reservoirs of environmental antibiotic resistance in terrestrial ecosystems. Owing to the inherently close relationship between plants and soil, there is a constant exchange of microbes between the phyllosphere and soil habitat, and thus the phyllosphere and soil could be important sources of ARGs for each other.^21–23^ However, because of the different conditions of plant surface and soil, the phyllosphere microbiota can be significantly different from the microbiota in the soils surrounding the plants.^24–26^ Due to the covering of hydrophobic cuticles and influences by ultraviolet radiation and plant metabolisms, the phyllosphere is an oligotrophic and stressful environment with a relatively lower microbial diversity compared to soil.^22^ Regardless of the plant species, phyllosphere microbiota from different locations is dominated by only a few bacteria phyla including Actinobacteria, Bacteroidetes, Firmicutes and Proteobacteria.^27,28^ By contrast, the soil is considered to harbour the most diverse microbial groups on Earth^29^ which are highly variable among different locations across the globe.^30^ In addition, a previous study found that some ARGs were not shared by the phyllosphere and surrounding soil^23^ suggesting that the evolutionary mechanisms of the phyllosphere and soil resistomes might be unique from each other. The comparative studies on the biogeographic patterns of the phyllosphere and soil resistomes, however, are lacking. This knowledge gap greatly impedes our ability in predicting the dissemination and evolution of antibiotic resistance in natural terrestrial ecosystems. Here, we conducted a large-scale investigation of the resistomes in the phyllosphere of predominant herbaceous vegetation types and soil from an >4000 km transect, which encompassed the major representative ecosystem types across eastern and northern Australia (i.e., forest, shrubland and grassland; Fig. 1). We aimed to address a set of questions that are critical to the understanding of environmental ARGs in natural terrestrial ecosystems including (i) How do the biogeographic patterns of resistomes in distinct environmental habitats of the phyllosphere and soil differ across a large spatial scale? (ii) What are the roles of spatial distance, co-occurring microbial communities and abiotic climatic and edaphic factors in shaping the biogeographic patterns of resistomes in these two habitats? We hypothesized that the different natures of the phyllosphere and soil environments would lead to unique responses of microbial communities to biotic and abiotic factors which would lead to distinct biogeographic patterns of resistomes for the two habitats.

## Results

### Large-scale atlas of the phyllosphere and soil resistomes

**Fig. 2:**
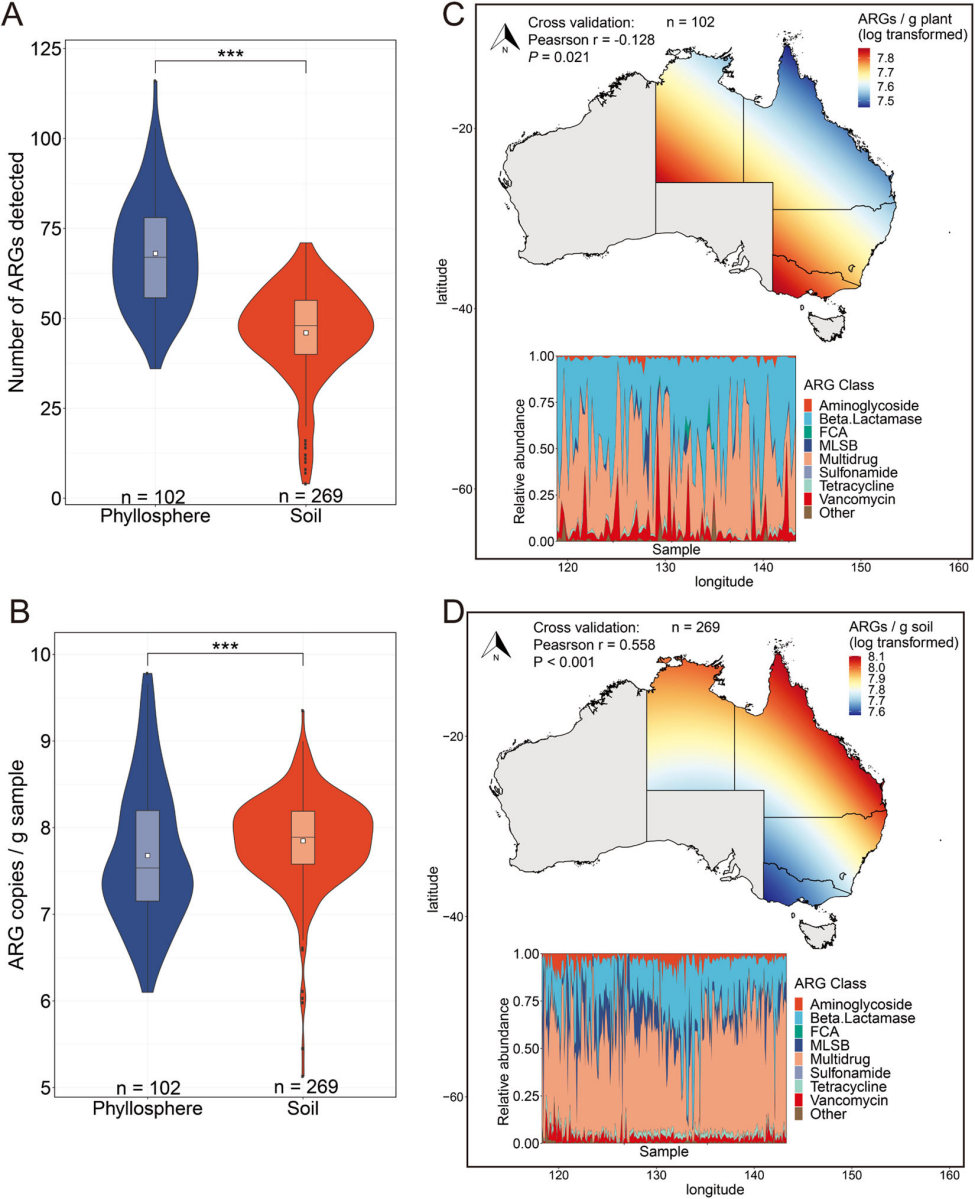
Characteristics of the ARGs detected in the phyllosphere and soil samples. Violin plots and box plots showing the numbers of detected ARGs (**A**) and the absolute abundance of all detected ARGs (**B**). Symbol (***) indicates Wilcoxon rank-sum test *P* < 0.001. White squares inside the box plots indicate the mean values. Large-scale atlas of the spatial distributions of ARG abundance in phyllosphere (**C**) and soil (**D**) across eastern and northern Australia. The compositions of ARGs detected in the samples are displayed in the bottom area charts. Samples showing in the area charts were ordered by ascending in the latitude of the sampling locations. FCA fluoroquinolone, quinolone, florfenicol, chloramphenicol and amphenicol resistance genes, MLSB Macrolide-Lincosamide-Streptogramin B resistance genes. Multidrug Efflux pump genes that resistant to multiple classes of antibiotics.

Using high-throughput quantitative PCR (HT-qPCR), we detected 264 and 268 ARGs in the 103 phyllosphere and 295 soil samples, respectively. More diverse (Fig. 2A) but less abundant (Fig. 2B) ARGs were detected in the phyllosphere than in soil (Wilcoxon rank-sum test, *P* < 0.001). The abundance of ARGs (copies/gram sample) ranged from 1.25 × 10^6^ to 5.98 × 10^9^ (with a median value of 3.43 × 10^7^) for the phyllosphere (Fig. S1) and 1.12 × 10^7^ to 2.26 × 10^9^ (with a median of 7.73 × 10^7^) for soil (Fig. S2). The ubiquitous (detected in all samples) and abundant (accounting for >40% of the total abundance) ARG classes were identified as dominant ARG classes. Multidrug resistance genes (i.e., efflux pumps that resistant to multiple classes of antibiotics), and beta-lactamase resistance genes were dominant in the phyllosphere resistome, which accounted for 85.26% of the total ARG abundance (Fig. 2C, insertion). The soil resistome was dominated by multidrug resistance genes which accounted for 54.25% of the total ARG abundance (Fig. 2D, insertion).

The resistomes in the phyllosphere and soil were well separated from each other as revealed by the nonmetric multidimensional scaling (NMDS) ordinations (ANOSIM *R* = 0.555, *P* < 0.001; Fig. S3). The distribution patterns of the ARG abundances were predicted using the kriging interpolation method. The results revealed that, for the phyllosphere resistome, more abundant ARGs were predicted in the southern and western parts of Australia than in the northern and eastern parts (Fig. 2C). An almost opposite trend of ARG abundance was observed for the soil resistome with the highest ARG abundance to be predicted in the northeast coastal regions of Australia (Fig. 2D). Similar trends in biogeographic patterns were observed for the dominant ARG class(es) in both phyllosphere (Fig. S4) and soil (Fig. S5).

### Characteristics of bacterial, fungal and protistan communities

**Fig. 3:**
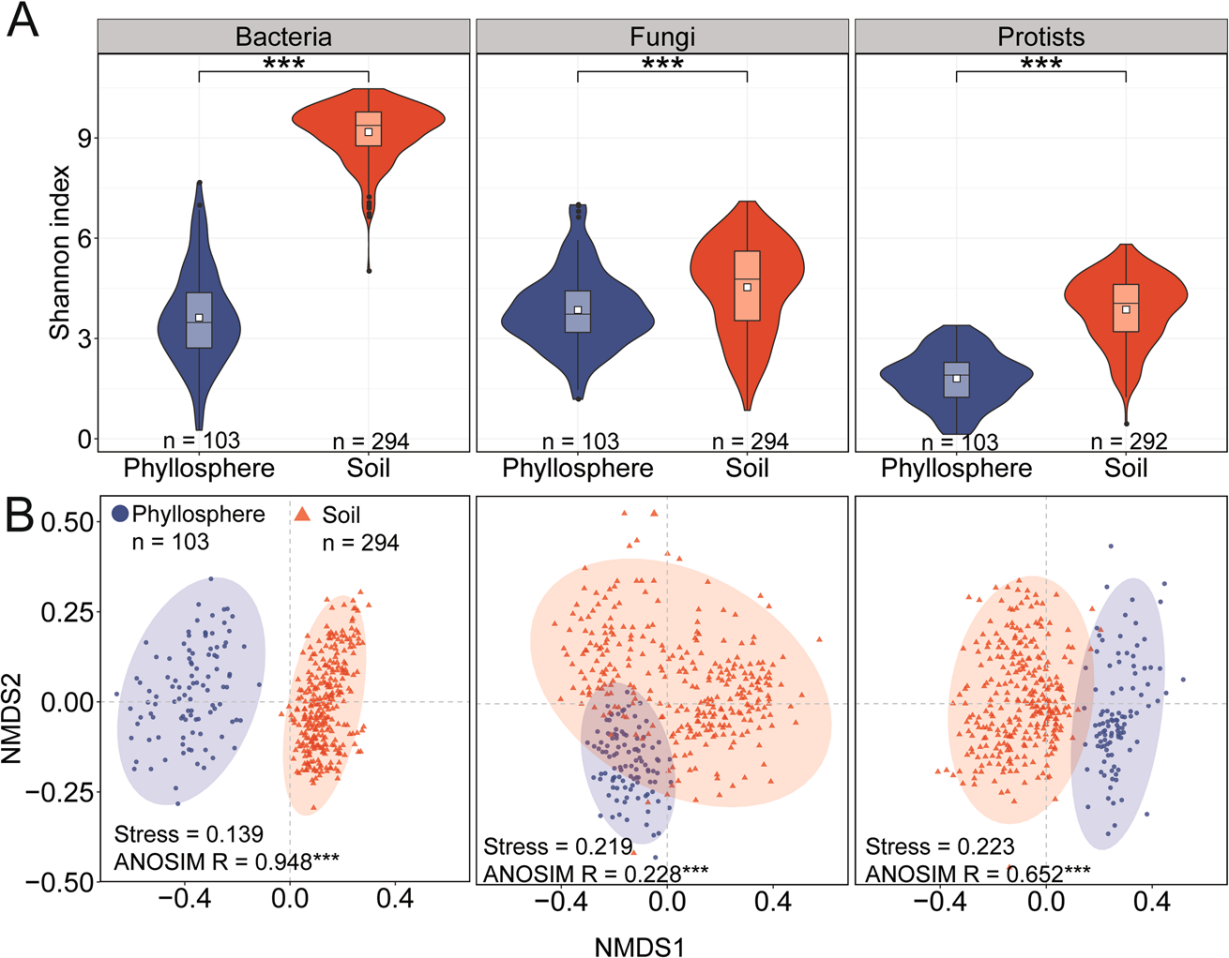
Characteristics of bacterial, fungal and protistan communities in phyllosphere and soils. **A** Violin plots and boxplots showing the Shannon index of bacterial, fungal and protistan communities in phyllosphere and soils. Symbol (***) indicates Wilcoxon rank-sum test *P* < 0.001. White squares inside the box plots indicate the mean values. **B** Nonmetric multidimensional ordinations of the microbial community structures at the OTU level among the phyllosphere and soil samples based on the Bray–Curtis distance. Symbol (***) indicates ANOSIM *P* < 0.001.

The high-quality sequences obtained from amplicon sequencing of the 16 S rRNA gene, the internal transcribed spacer (ITS) region, and the 18 S rRNA gene were clustered into 40,997 bacterial, 29,525 fungal and 23,545 protistan operational taxonomic units (OTUs) at 97% similarity level, respectively. The alpha-diversity of all the three investigated microbial communities in soil was significantly higher than that in the phyllosphere (Wilcoxon rank-sum test, *P* < 0.001; Figs. 3A and S6). Clear separations between the microbial communities in the phyllosphere and soil were revealed by the NMDS ordinations (bacterial community: ANOSIM *R* = 0.948, *P* < 0.001, fungal community: ANOSIM *R* = 0.228, *P* < 0.001; protistan community: ANOSIM *R* = 0.652, *P* < 0.001; Fig. 3B). Proteobacteria was the most abundant bacterial phylum in the phyllosphere, which accounted for 79.38% of the 16 S rRNA gene sequences, followed by Actinobacteria (9.61%; Fig. S7A). Actinobacteria was the most abundant bacterial phylum in the soil which accounted for 37.73% of the 16 S rRNA gene sequences. The second most abundant bacterial phylum in soil was Proteobacteria which accounted for 24.86% of the 16 S rRNA gene sequences (Fig. S7A). The majority of the ITS region sequences belonged to the fungal phylum Ascomycota, which accounted for 92.32% and 78.59% of total sequences in the phyllosphere and soil, respectively (Fig. S7B). The three most abundant protistan phyla in the phyllosphere were Metazoa (54.73%) of the kingdom Opisthokonta, and Ciliophora (20.78%), and Apicomplexa (13.91%) of the kingdom AlveolataM (Fig. S7C). For soil, Metazoa (44.20%) was the most abundant protistan phylum, followed by Cercozoa (29.56%) of the kingdom Rhizaria and Ciliophora (25.24%; Fig. S7C). Analysis of the difference in the abundances of bacteria in the phyllosphere and soil showed that the bacterial abundance in soil was significantly higher than that in the phyllosphere (Wilcoxon rank-sum test *P* < 0.001; Fig. S8).

### Distance-decay relationships for the resistomes and microbial communities and spatial configuration of the sampling sites

**Fig. 4:**
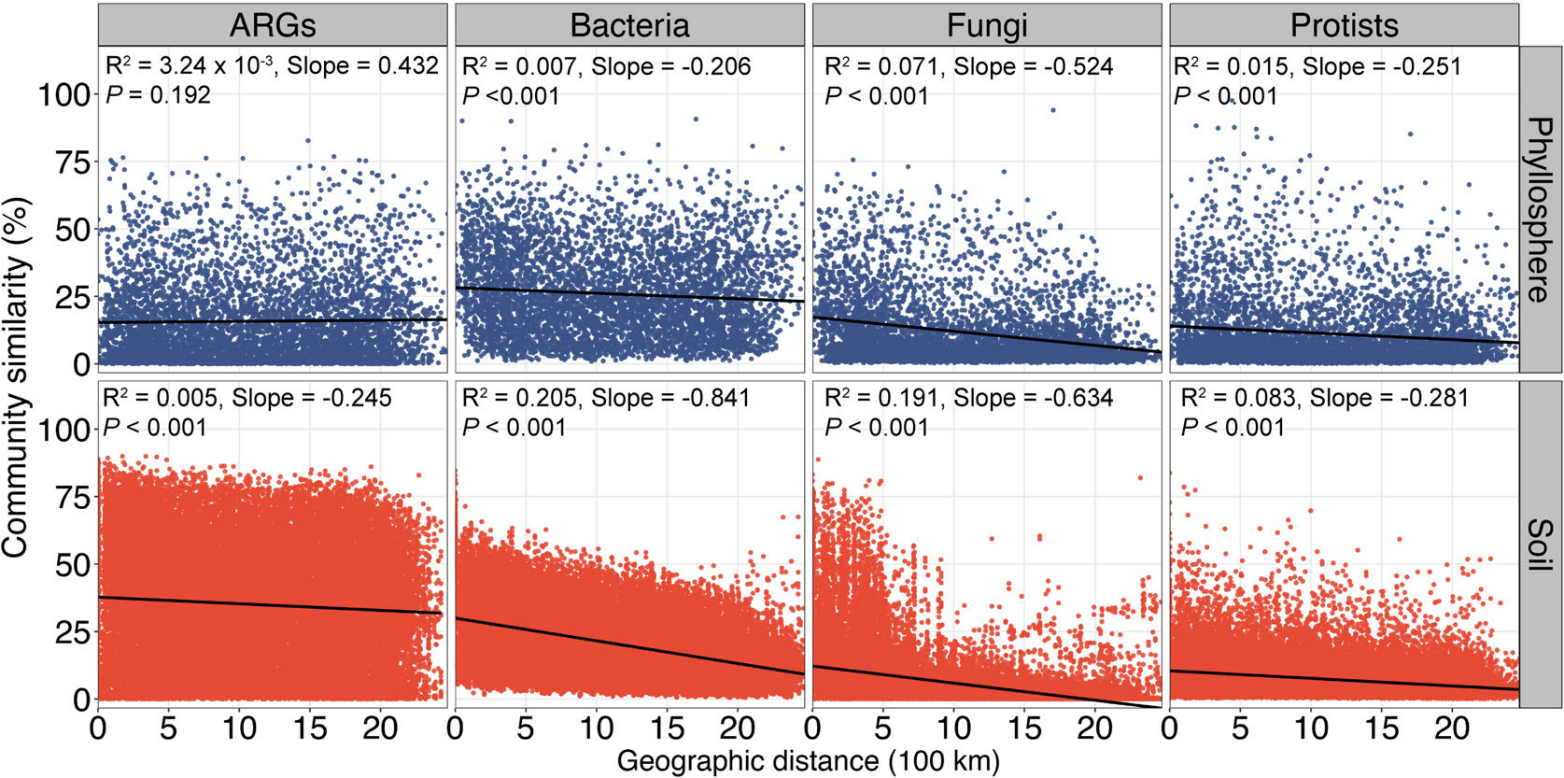
Distance-decay relationships for ARGs, bacterial, fungal and protistan communities at the OTU level in the phyllosphere and soil samples. Symbol (***) indicates ordinary least regression *P* < 0.001. Significant correlations (*P* < 0.05) and no significant correlation (*P* > 0.05) are shown in solid lines and dashed line, respectively.

The distance-decay relationships (DDRs) for ARGs and microbial communities in the phyllosphere and soil were estimated. For the phyllosphere resistome, no DDR was observed for neither the entire resistome (Fig. 4) nor the dominant ARG classes (Fig. S9). For the soil resistome, although DDR was significant (*P* < 0.001) for both the entire resistome (Fig. 4) and the dominant ARG class (Fig. S9), the effect sizes were remarkably small (*R*^2^ < 0.1), indicating weak decay of resistome similarity with geographic distance. Significant (*P* < 0.001) DDRs were observed for all three investigated microbial communities in both the phyllosphere and soil (Fig. 4) with the soil microbial communities having larger effect sizes (bacterial community: *R*^2^ = 0.205; fungal community: *R*^2^ = 0.191; protistan community: *R*^2^ = 0.083) than the corresponding phyllosphere communities (bacterial community: *R*^2^ = 0.007; fungal community: *R*^2^ = 0.071; protistan community: *R*^2^ = 0.015). In addition, the slopes of distance-decay curves were steeper for the soil microbial communities (bacterial community: slope = −0.841; fungal community: slope = −0.191; protistan community: slope = −0.083,) than the corresponding phyllosphere communities (bacterial community: slope = −0.206; fungal community: slope = −0.524; protistan community: slope = −0.281).

Variations in the spatial configuration among the sampling sites were explored by the Moran’s eigenvector maps (MEMs).^31^ The eigenvalues and Moran’s *I* of the MEMs for the sampling sites are summarised in Fig. S10A and Table S1, respectively. The MEMs revealed that our sampling sites showed a significant clustering pattern (autocorrelation) at a large-scale. The autocorrelations of the sampling sites get weaker with the decrease in spatial scale. Examples of some significant MEMs (MEMs with positive eigenvalues and significant Moran’s *I*) are mapped in geographic space (Fig. S10B).

### Drivers of ARG abundance in the phyllosphere and the soil environments

Spearman’s correlation analysis revealed that ARG abundance in the phyllosphere was not only significantly (*P* < 0.001) positively correlated with the bacterial community composition, but also was significantly (*P* < 0.05) correlated with the community compositions of fungal and protistan communities. However, none of the investigated abiotic factors was significantly correlated with the phyllosphere ARG abundance (Table 1). In contrast, soil ARG abundance was significantly (*P* < 0.05) positively correlated with abiotic factors including mean annual temperature (MAT) and mean annual precipitation (MAP), as well as soil total carbon (TC) and total nitrogen (TN), but not with the compositions of any investigated microbial communities (Table 1). Similar relationships between the abundances of ARGs of the dominant class(es) with these biotic and abiotic factors were found for both the phyllosphere and soil samples (Table S2).

**Table 1:** Spearman’s correlation between ARG abundance and selected biotic and abiotic factors.

Microbial community compositions			Climatic factors		Edaphic factors			
Bacterial NMDS1	Fungal NMDS1	Protistan NMDS1	MAT (°C)	MAP (mm)	TC (%)	TN (%)	pH	
**0.636**	**−0.333**	**0.281**	−0.060	−0.128	−0.088	0.002	0.084	**Phyllosphere**
***P*** **< 0.001**	***P*** = **0.001**	***P*** = **0.004**	*P* = 0.546	*P* = 0.201	*P* = 0.379	*P* = 0.981	*P* = 0.403	
**0.044**	0.021	0.106	**0.177**	**0.203**	**0.217**	**0.232**	0.041	**Soil**
***P* = 0.472**	*P* = 0.731	*P* = 0.086	***P*** = **0.004**	***P*** = **0.001**	***P*** < **0.001**	***P*** < **0.001**	*P* = 0.501	

Significant correlations (*P* < 0.05) are indicated as bold fonts.

To further explore how space differently impacts the observed distinct biogeographic patterns of the soil and phyllosphere resistomes, distance-based redundancy analysis (dbRDA) was conducted with the most influential MEMs (top five largest axes of the MEMs) and edaphic as well climatic factors of each sampling sites as explanatory variables. The dbRDA results reveal that the soil resistome was not only significantly influenced by edaphic factors but also significantly impacted by changes in spatial configurations and corresponding changes in MAT and MAP (Fig. S11A). However, the phyllosphere resistome was only significantly impacted by spatial configuration at large-scale (MEM1) and MAP (Fig. S11B).

**Fig. 5:**
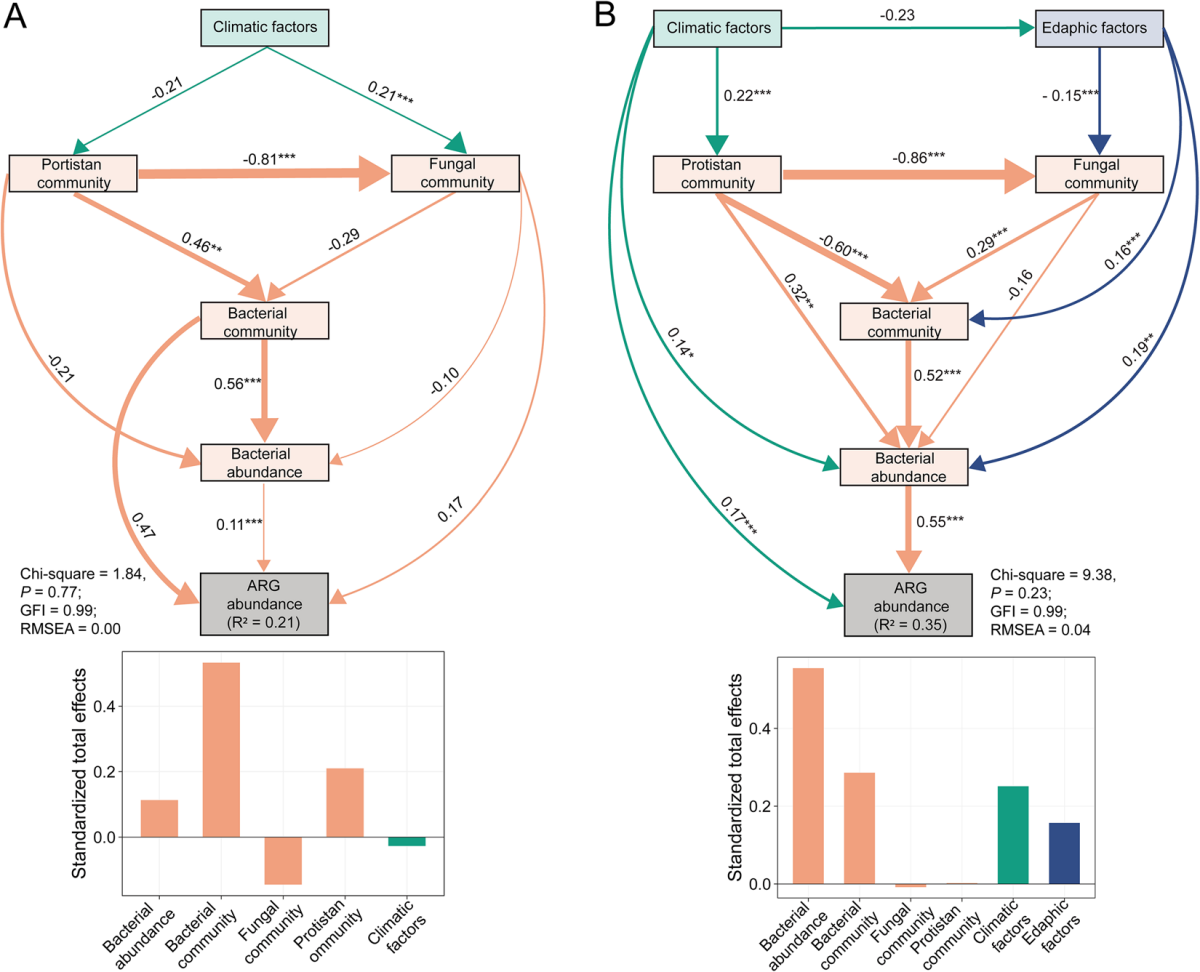
Drivers of ARG abundances in phyllosphere and soils. Structure equation models (SEMs) illustrating the direct and indirect effects of multiple factors on the variations in ARG abundance in phyllosphere (**A**) and soils (**B**). The width of the arrows is proportional to the strength of path coefficients shown by the numbers adjacent to the arrows. *R*^2^ indicates the proportion of variations in ARG abundance explained by the models. Symbol (*) indicates < 0.05; symbol (**) indicates *P* < 0.01; symbol (***) indicates *P* < 0.001. Standardised effects were derived from the SEMs. GFI goodness of fit index, RMSEA root mean square error of approximation.

The direct and indirect contributions of different factors to the variations in the ARG abundances were further illustrated with structural equation models (SEMs), which explained 21 and 35% of the variations in the ARG abundance for the phyllosphere and the soil resistomes, respectively (Fig. 5). For the phyllosphere resistome, bacterial and fungal community compositions and bacterial abundance had direct impacts on ARG abundance. Fungal and protistan community compositions had indirect impacts on ARG abundance through impacting bacterial community composition and abundance. However, climatic factors only had a weak impact on phyllosphere ARG abundance through influencing fungal and protistan community compositions (Fig. 5A). For the soil resistome, the only biotic factor directly impacting the ARG abundance was the abundance of bacteria. Fungal and protistan community compositions only had indirect impacts on ARG abundance through impacting on bacterial abundance. Compared to fungal and protistan communities, climatic and edaphic factors had relatively strong impacts on the variation in soil ARG abundance. Particularly, climatic factors had both direct and indirect impacts on the variation in soil ARG abundance (Fig. 5B).

## Discussion

### Contrasting resistome profiles in the phyllosphere and soil

In this study, we presented a large-scale investigation into the phyllosphere and soil resistomes across a variety of natural ecosystems. Our results revealed contrasting resistome profiles in the phyllosphere and soil with the phyllosphere harbouring more diverse but less abundant ARGs than soil (Fig. 2). A possible reason for the higher ARG diversity in the phyllosphere than soil is that, as an open habitat, the phyllosphere is subject to wide sources of microorganisms, and ARGs, including those transmitted by insects and aerosols.^21^ A previous study reported that for phyllosphere and soil from the same location, the types of phyllosphere specialist ARGs are more than soil specialist ARGs.^23^ The differences in the abundance of ARGs in the phyllosphere and soil may mainly be attributed to the differences in the background bacteria abundances, which are the potential hosts of ARGs. The comparison of 16 S rRNA gene copy numbers revealed that the bacterial abundance in the soil was more than one order of magnitude higher than that in the phyllosphere (Fig. S8). In addition, the accumulation of ARGs in soil may also contribute to the significantly higher ARG abundance in soil than in the phyllosphere.^32^ Therefore, the ARGs transmitted from the phyllosphere may also accumulate in soil and contribute to the increase in soil ARG abundance. We observed similar trends of ARG abundances of the dominant ARG class(es) and the entire resistomes in both the phyllosphere and soil habitats, which indicate that there might be predictable biogeographic patterns of environmental ARGs in natural ecosystems. The biogeographic patterns of ARG abundance in the phyllosphere and soil were almost opposite, implying that the resistome profiles in the above-ground plant surface habitat and underground soil habitat might be differently shaped by biotic and abiotic factors.

### The soil resistome varies more significantly across space than the phyllosphere resistome

The MEMs revealed that there were autocorrelation patterns of our sampling sites at a large spatial scale, indicating that geographic distance might play a role in driving the community structures for our samples. The distance-decay relationship is a fundamental ecological pattern to describe the relationship between community similarity and geographic distance.^33,34^ In this study, we observed significant (*P* < 0.001) DDRs for ARGs in soil but not in the phyllosphere. The effect sizes of the DDRs for the microbial communities in the phyllosphere were substantially lower than those of the microbial communities in surrounding soils (Fig. 4) and reported in other natural soil ecosystems.^35^ These results demonstrated that compared to those in soil, the phyllosphere microbial communities and resistomes vary less significantly across space which is further supported by the dbRDA results (Fig. S11). The distinct impacts of spatial distance on the phyllosphere and soil resistomes could be derived from the differences in microbial dispersal limitation.^36^ The phyllosphere is an environment subjected to the fluctuations of the surrounding environment, for example, changes in wind and rainfall, which can facilitate the transmission of microbes in the phyllosphere from site to site.^22^ The low dispersal limitation in the phyllosphere environment would lead to relatively similar microbial communities, and thus similar resistome profiles, among sites spatially far from each other.^37^ Different from the phyllosphere, the soil is a relatively stable environment in which microorganisms are subject to slow-changing processes.^38,39^ Therefore, soil microorganisms would experience high dispersal limitation which would result in more similar resistome profiles between sites closer to each other than those further apart.^40^

### Distinct drivers of the biogeographic patterns of the phyllosphere and soil resistomes

Our results revealed that the ARG abundance in the phyllosphere was significantly correlated with the community compositions of bacterial, fungal and protistan communities but not with any of the investigated abiotic factors. While for soil, the ARG abundance was more apparently influenced by abiotic factors, including MAT, MAP, soil TC and TN, than by microbial community compositions. These results suggest that microbial interactions and abiotic climatic and edaphic factors might be the main drivers shaping the phyllosphere and soil resistomes, respectively. These contrasting responses could be potentially derived from the intrinsic differences of the two habitats. Compared to the soil resistome, the phyllosphere resistome and bacterial community were less heterogeneous across geographic locations (Fig. 4). Therefore, for the phyllosphere resistome the impacts of abiotic factors, which shift with geography, would be relatively weak and the roles of inter-kingdom interactions among microbial communities would become more important. The bacteria-fungal antagonism is a kind of very important inter-kingdom interaction that can lead to the production of considerable amounts of antibiotics by some bacterial and fungal cells which select against bacteria lacking ARGs.^8,11^ Another inter-kingdom interaction that could greatly influence the expression of ARGs by bacterial cells is the predation of bacteria by protists, which could pose substantial biotic stress to the bacterial community.^41^ The expression of some ARGs (for example, some efflux pump genes) can increase the general tolerance of bacteria to stresses other than the presence of antibiotics. Under protistan predation, bacterial cells would increase the expression of ARGs as a strategy to survive, which may partially explain the correlations between phyllosphere ARG abundance with the protistan community.^42^ On the contrary, the soil is a stable habitat with relatively high spatial heterogeneity.^38,39^ In soil habitat, the microbial interactions and gene expressions are underpinned by endemic abiotic factors.^39,43^ A potential explanation for the observed significant correlations between MAT and MAP with soil ARG abundance could be that genotypic alternations of bacterial cells induced by environmental stresses (e.g. temperature extremes and water shortage) are similar to those induced by the action of antibiotics.^44^ The availability of soil nutrients could also be closely related to the ARG abundance by influencing the types of microbial interactions.^45^ The stress gradient hypothesis assumes that an increase in nutrient availability would enhance the frequency of negative microbial interactions which may induce antibiotic production.^45^ Such a relationship between growth-limiting nutrient availability and the direction of microbial interactions could potentially explain the observed positive relationships between soil TC and TN with soil ARG abundance.

Nevertheless, it should be noted that there are some potential caveats in the interpretation of our results. The plant host species were not considered in the present study, but it has been identified as a potential influencing factor of phyllosphere resistomes.^46,47^ In addition, the presence of stochastic events and persistent microbial trait variations were not addressed, but those factors could play a role in shaping microbial community assembly^1^ and thus resistome profiles. These limitations together with the potential sampling effects could explain why >60% of the variations in the ARG abundance remain unexplained for both the phyllosphere and the soils (Fig. 5). It is impractical to address all potential variables for a large-scale survey, and this is inconsistent with previous large-scale microbial studies which explained 10–50% of the variations in microbial patterns.^1,48–50^ Despite these limitations, our results revealed that the biotic interactions among bacterial, fungal and protistan communities and abiotic factors, including MAT, MAP, soil TC and TN, differently drive the variations in the phyllosphere and soil resistome profiles.

## Conclusion

In this study, we provided a systematic investigation into the biogeographic patterns of the phyllosphere and soil resistomes in natural ecosystems. We found contrasting biogeographic patterns of the phyllosphere and soil resistomes, which may derive from the distinct impacts of biotic and abiotic factors on the microbial communities in these two environmental habitats. The phyllosphere resistome, which was not sensitive to the changes in abiotic factors across space, was mainly impacted by the inter-kingdom interactions among bacterial, fungal and protistan communities. By contrast, MAT, MAP and soil nutrients were the main drivers shaping the soil resistome. Our results imply that the characteristics of microbial habitat are critical in underpinning the biogeographic patterns of resistomes in natural ecosystems. Therefore, it is necessary to consider the intrinsic differences in habitats when predicting the evolution and spread of environmental ARGs.

## Materials and methods

### Sampling campaign

Herbaceous plant and soil samples were collected during May 2019 from a total of 118 locations across eastern and northern Australia with minimal anthropogenic impacts (Fig. 1). MAT and MAP of the sampling sites ranged from 12.93–26.54 °C and 254–1901 mm, respectively. At each location, we established a 20 × 20 m plot in which three subplots (1 × 1 m) were established on the most predominant herbaceous vegetation type. The aerial parts of the herbaceous plants were collected using sterilized scissors to form a composite plant sample for each plot. Three independent soil samples were collected from each plot by mixing five soil cores (0–10 cm) into one composite sample. Our large-scale sampling collected a total of 103 herbaceous plant samples and 295 soil samples, which were transported to the laboratory on dry ice. The plant samples were immediately used for DNA extraction from the phyllosphere microbes. The soil samples were sieved through a 2 mm mesh and divided into two portions. One portion was air-dried for soil physicochemical characterization, and the other portion was stored at −20 °C before molecular analyses.

### Soil physicochemical characterization and climatic data acquisition

Soil physicochemical properties including soil TC, TN and soil pH were determined using standardized protocols.^51^ Briefly, soil TC and TN were determined using the Dumas combustion method on a LECO EP628 analyser (LECO Australia Pty, Ltd, Baulkham Hills, NSW, Australia). Soil pH was measured in 1:5 soil water suspension using an Orion Star A215 pH/Conductivity Benchtop Multiparameter Metre (Thermo Scientific Inc., Waltham, MA, USA). Detailed information on soil physicochemical properties is provided in Supplementary File 2. Estimates of MAT and MAP at 30 arc seconds (~1 km^2^) were obtained from the WorldClim database (https://worldclim.org/).^52^ MAT and MAP values of each sampling location are shown in Supplementary File 3.

### Molecular analyses

Plant phyllosphere genomic DNA and soil genomic DNA were extracted using the DNeasy Power Soil kit (QIAGEN). Pty. Ltd, Chadstone Centre, VIC, Australia) *as per* the manufacture’s instruction. The plant sample preparation for phyllosphere genomic DNA extraction was conducted according to a previous method.^23,53^ Briefly, around 5 g leaf tissue was weighted into a conical flask (25 m mL) containing 100 mL of 0.01 M autoclaved phosphate-buffered saline (PBS). The mixture was sonicated for 7 min and shaken at 180 rpm at 30 °C for 1 h. The obtained solution was filtered with nylon gauze and then a 0.22 µM cellulose membrane. The cellulose membrane was cut into pieces and used for DNA extraction. ARG abundance was determined using an HT-qPCR technique on the Wafergen SmartChip Real-Time Platform (Fremont, CA, USA). The HT-qPCR array contains primers targeting 285 ARGs that confer resistance to all major classes of antibiotics commonly used in humans and animals and the 16 S rRNA gene. Detailed information on the primers is provided in Supplementary File 4. Amplification of ARGs was conducted in a 100 nL reaction system. The thermal cycle for the amplification was as follows: and initial enzyme activation at 95 °C for 10 min, followed by 40 cycles of 95 °C for 30 s and 60 °C for 30 s. The melting process was automatically generated by the Wafergen SmartChip software (V2.7.0). Only well data with a single melting peak and an amplification efficiency within 1.8–2.2 were retained. A cycle threshold (C_T_) value of 31 was used as the detection limit. The relative copy numbers of ARGs were calculated according to the following equation: Relative gene copy number = 10^(31-Ct)/ (10/3).^^54,55^ The relative copy numbers of ARGs were transformed to absolute copy numbers by normalization with 16 S rRNA gene copy number quantified using a Bio-Rad CFX384 Real-Time PCR detection system (Bio-Rad, Hercules, USA). The 20 μl PCR reaction system for 16 S rRNA gene quantification contained 10 μl Sensimix SYBR NO-ROX reagent (Bioline, London, UK), 0.8 μl each primer (10 μM) and 2 μl DNA template. The amplicon condition was the same as that for the amplification of ARGs. It should be noted that because the surface areas of the plant cannot be accurately measured and the mass of the herbaceous plants is marginal compared to their surface area, we reported the ARG abundance in both the phyllosphere samples as ARG copies/gram plant tissue according to previous studies.^23,56^ The ARG classes that are ubiquitously (detected in all samples) and abundant (accounted for >40% of total ARG abundance) were identified as dominant ARG classes in the phyllosphere and soil samples.

The 16 S rRNA gene, the ITS region, and the 18 S rRNA gene were amplified for the characterization of bacterial, fungal and protistan communities, respectively, on an Illumina Miseq EP300 platform at Shanghai Majorbio Bio-pharm Technology Co., Ltd. Primer sets used for the amplicon sequencings were 515FmodF and 806RmodR for 16 S rRNA gene,^57^ ITS1F and ITS2R for ITS region^58^ and TAReukFWD1F and TAReukREV3R for 18 S rRNA gene.^59^ Raw sequencing reads were merged using FLASH^60^ and quality filtered according to the following criteria: raw pair-end reads containing more than three ambiguous nucleotides, reads with an average quality score <20, short reads (< 100 nt) and the barcode was discarded. The generated high-quality sequences were analysed and processed using Quantitative Insight into Microbial Ecology (QIIME).^61^ OTUs were picked at 97% similarity level using UCLUST.^62^ Singleton OTUs and chimeric sequences, chloroplast and mitochondrial OTUs were discarded from the OTU table before downstream analysis. Taxonomic classification of OTUs was conducted using the SILVA database for the 16 S rRNA gene,^63^ UNITE database 8.0 for the ITS region,^64^ and Protists Ribosomal Reference (PR2) database for the 18 S rRNA gene PR2.^65^

### Statistical analyses

All statistical analyses were conducted in the R platform (V3.6.3; http://www.r-project.org/) and visualised using the ‘ggplot2’ package.^66^ Statistical results were considered significant at *P* < 0.05 level. *P* values for multiple comparisons were corrected using the Benjamini–Hochberg procedure. The differences in the numbers of the detected ARGs, the abundance of the detected ARGs, and microbial alpha-diversity indices between the phyllosphere and the soil samples were determined using Wilcoxon rank-sum test with the ‘stats’ package.^67^ Log-transformed ARG abundances in all plant phyllosphere and soil samples were shown in heatmaps constructed with the ‘pheatmap’ package.^68^ The relationships of resistomes (based on ARG abundance) and microbial communities (at the OTU level) in the phyllosphere and the soils were visualised by NMDS ordinations based on Bray–Curtis distance with the ‘vegan’ package.^69^ The significant differences in the resistome compositions and microbial beta-diversities between the phyllosphere and the soils were determined using PERMANOVA analysis with ANOSIM function in the ‘vegan’ package.^69^ The spatial distributions of the abundance of all detected ARGs and the ARGs belonging to the dominant ARG classes in the phyllosphere and the soils were estimated using the kriging interpolation method with the ‘automap’ package.^70^ Cross-validations of the predicted maps were based on the Pearson correlation between the prediction values and the observed values of the ARG abundances for the same site. MEMs for the sampling sites were constructed using the ‘adespatial’ package^71^ based on the spatial weight matrix defined using the ‘spdep’ package.^72^ Each MEM eigenvalue quantified a spatial autocorrelation at a given spatial scale. The eigenvalues of the MEMs are automatically sorted from large-scale to fine-scale. Moran’s *I* for the spatial autocorrelation were calculated with 999 permutations using the ‘adespatial’ package.^71^ MEMs with a positive eigenvalue and a significant Moran’s*I*were considered to describe significant spatial autocorrelations.^73^ DDRs for the resistomes and the microbial community compositions were calculated as the slopes of the OLS regression relationships between the community similarity (1-Bray–Curtis dissimilarity matrices) calculated with the ‘vegan’ package^69^ and geographic distance calculated with the ‘sp’ package.^74^ Spearman’s correlations between the ARG abundances and NMDS1 values of bacterial, fungal and protistan communities as well as MAT, MAP, soil TC, TN and pH were explored with the ‘stats’ package.^67^ dbRDA to explore the impacts of changes in spatial configurations and edaphic as well as climatic factors on the soil and phyllosphere resistomes were conducted with the ‘vegan’ package.^69^

### Structure equation modelling

The SEMs^75^ were conducted using IBM SPSS Amos (V25; Amos Development Corporation, Chicago, IL, USA) to evaluate the direct and indirect impacts of a variety of biotic and abiotic factors on variations in ARG abundance in the phyllosphere and the soils. A prior model was established based on hypothesized effects and relationships among the drivers of the ARG abundances (Fig. S12). NMDS1 values of the NMDS analysis were used for the representation of bacterial, fungal and protistan community structures in the SEMs. Bacterial abundance was estimated by the copy numbers of 16 S rRNA genes. The overall goodness of fit for the SEMs was determined by multiple criteria: chi-square test *P* > 0.05, goodness of fit index (GFI) > 0.90 and root mean square error of approximation (RMSEA) < 0.08.^76^

## Supplementary information

Supplementary Information File1

Supplementary Information File2

Supplementary Information File3

Supplementary Information File4

## Data Availability

The raw sequence data reported have been deposited in the National Centre for Biotechnology Information (NCBI) Sequence Read Archive (SRA) under the accession number PRJNA659980 (soil and plant samples titled with S and G).
