# Supplementary material for: Biotic and abiotic factors distinctly drive contrasting biogeographic patterns between phyllosphere and soil resistomes in natural ecosystems
Source: ISME Commun. 2021 Apr 14;1:13. doi: 10.1038/s43705-021-00012-4 (PMC9645249; doi:10.1038/s43705-021-00012-4)
Supplement: Supplementary file 1 — Supplementary Information File1 [file 43705_2021_12_MOESM1_ESM.docx]

**Supplementary information**

Biotic and abiotic factors distinctly drive contrasting biogeographic patterns between phyllosphere and soil resistomes in natural ecosystems

**Running title:**

Distinct biogeographic patterns of phyllosphere and soil resistomes in natural ecosystems

Zhen-Zhen Yan ^1^, Qing-Lin Chen ^1*^, Chao-Yu Li ^1^, Bao-Anh Thi Nguyen ^1^, Yong-Guan Zhu ^2^, Ji-Zheng He ^1^, Hang-Wei Hu ^1*^

^1^ School of Agriculture and Food, Faculty of Veterinary and Agricultural Sciences, The University of Melbourne, VIC, 3010, Australia.

^2^ Key Laboratory of Urban Environment and Health, Institute of Urban Environment, Chinese Academy of Sciences, 1799 Jimei Road, Xiamen, 361021, China

*Corresponding authors:

[qinglin.chen@unimelb.edu.au](mailto:qinglin.chen@unimelb.edu.au) (Q-L, Chen);

[hang-we.hu@unimelb.edu.au](mailto:hang-wei.hu@unimelb.edu.au) (H-W, Hu).

**Summary of contents**

Supplementary file 1: Table S1; Table S2; Fig. S1 – S12, .docx file.

Supplementary file 2: list of soil properties, .xlsx file.

Supplementary file 3: list of climatic factors, .xlsx file.

Supplementary file 4: Information of primers used for the HT-qPCR quantification of ARGs, xlsx file.

**Table S1** Moran’s *I* for each axis of the Moran’s eigenvector maps.

| **Test** | **Obs** | **Std.Obs** | **Alter** | ***P*-value** |
| --- | --- | --- | --- | --- |
| MEM1 | 1.016073 | 12.47069 | greater | 0.001 |
| MEM2 | 1.007145 | 12.12525 | greater | 0.001 |
| MEM3 | 1.001725 | 12.35922 | greater | 0.001 |
| MEM4 | 0.994288 | 11.64666 | greater | 0.001 |
| MEM5 | 0.985704 | 12.24134 | greater | 0.001 |
| MEM6 | 0.983526 | 12.00557 | greater | 0.001 |
| MEM7 | 0.961351 | 11.57866 | greater | 0.001 |
| MEM8 | 0.948027 | 11.35398 | greater | 0.001 |
| MEM9 | 0.936125 | 11.57989 | greater | 0.001 |
| MEM10 | 0.92354 | 11.62942 | greater | 0.001 |
| MEM11 | 0.920674 | 11.35698 | greater | 0.001 |
| MEM12 | 0.911244 | 11.17902 | greater | 0.001 |
| MEM13 | 0.89356 | 11.11894 | greater | 0.001 |
| MEM14 | 0.876649 | 10.92835 | greater | 0.001 |
| MEM15 | 0.869353 | 10.75226 | greater | 0.001 |
| MEM16 | 0.861344 | 10.34135 | greater | 0.001 |
| MEM17 | 0.836387 | 10.1415 | greater | 0.001 |
| MEM18 | 0.826728 | 10.04655 | greater | 0.001 |
| MEM19 | 0.820696 | 10.29073 | greater | 0.001 |
| MEM20 | 0.79205 | 10.22037 | greater | 0.001 |
| MEM21 | 0.772148 | 9.266583 | greater | 0.001 |
| MEM22 | 0.764483 | 9.356284 | greater | 0.001 |
| MEM23 | 0.731543 | 8.816545 | greater | 0.001 |
| MEM24 | 0.71979 | 9.046406 | greater | 0.001 |
| MEM25 | 0.7119 | 8.715064 | greater | 0.001 |
| MEM26 | 0.689276 | 8.318112 | greater | 0.001 |
| MEM27 | 0.672944 | 8.221434 | greater | 0.001 |
| MEM28 | 0.645027 | 7.753772 | greater | 0.001 |
| MEM29 | 0.605278 | 7.337623 | greater | 0.001 |
| MEM30 | 0.603011 | 7.550533 | greater | 0.001 |
| MEM31 | 0.577786 | 6.950992 | greater | 0.001 |
| MEM32 | 0.562784 | 6.580494 | greater | 0.001 |
| MEM33 | 0.552549 | 6.853402 | greater | 0.001 |
| MEM34 | 0.499921 | 6.037462 | greater | 0.001 |
| MEM35 | 0.478008 | 5.671883 | greater | 0.001 |
| MEM36 | 0.435202 | 5.394886 | greater | 0.001 |
| MEM37 | 0.421335 | 5.396996 | greater | 0.001 |
| MEM38 | 0.413466 | 5.156617 | greater | 0.001 |
| MEM39 | 0.386612 | 4.84586 | greater | 0.001 |
| MEM40 | 0.36228 | 4.292384 | greater | 0.001 |
| MEM41 | 0.328751 | 4.188434 | greater | 0.001 |
| MEM42 | 0.297244 | 3.709412 | greater | 0.001 |
| MEM43 | 0.281599 | 3.620684 | greater | 0.001 |
| MEM44 | 0.263598 | 3.338538 | greater | 0.001 |
| MEM45 | 0.243117 | 3.190355 | greater | 0.004 |
| MEM46 | 0.211396 | 2.638925 | greater | 0.007 |
| MEM47 | 0.186026 | 2.423659 | greater | 0.011 |
| MEM48 | 0.169185 | 2.135982 | greater | 0.019 |
| MEM49 | 0.151682 | 2.040945 | greater | 0.023 |
| MEM50 | 0.117974 | 1.478805 | greater | 0.07 |
| MEM51 | 0.108437 | 1.353437 | greater | 0.089 |
| MEM52 | 0.102945 | 1.346525 | greater | 0.097 |
| MEM53 | 0.061933 | 0.838264 | greater | 0.195 |
| MEM54 | 0.019531 | 0.362509 | greater | 0.364 |
| MEM55 | 0.000863 | 0.095527 | greater | 0.45 |
| MEM56 | -0.00283 | 0.128715 | greater | 0.439 |
| MEM57 | -0.02751 | -0.20758 | greater | 0.572 |
| MEM58 | -0.06538 | -0.70861 | greater | 0.759 |
| MEM59 | -0.07417 | -0.74619 | greater | 0.766 |
| MEM60 | -0.10442 | -1.24919 | greater | 0.899 |
| MEM61 | -0.10959 | -1.25883 | greater | 0.893 |
| MEM62 | -0.12547 | -1.38193 | greater | 0.919 |
| MEM63 | -0.15402 | -1.72185 | greater | 0.956 |
| MEM64 | -0.16513 | -1.87923 | greater | 0.966 |
| MEM65 | -0.1682 | -1.88269 | greater | 0.971 |
| MEM66 | -0.19329 | -2.27855 | greater | 0.987 |
| MEM67 | -0.22187 | -2.78938 | greater | 0.996 |
| MEM68 | -0.23928 | -2.71931 | greater | 0.998 |
| MEM69 | -0.25959 | -2.89872 | greater | 1 |
| MEM70 | -0.29787 | -3.43362 | greater | 1 |
| MEM71 | -0.29973 | -3.52491 | greater | 1 |
| MEM72 | -0.31648 | -3.75259 | greater | 1 |
| MEM73 | -0.32737 | -3.86406 | greater | 1 |
| MEM74 | -0.36885 | -4.48233 | greater | 1 |
| MEM75 | -0.37414 | -4.45334 | greater | 1 |
| MEM76 | -0.40751 | -4.8033 | greater | 1 |
| MEM77 | -0.40969 | -4.98806 | greater | 1 |
| MEM78 | -0.43541 | -5.07918 | greater | 1 |
| MEM79 | -0.44637 | -5.27993 | greater | 1 |
| MEM80 | -0.45432 | -5.43351 | greater | 1 |
| MEM81 | -0.47969 | -5.65986 | greater | 1 |
| MEM82 | -0.48449 | -5.54774 | greater | 1 |
| MEM83 | -0.50829 | -5.92709 | greater | 1 |
| MEM84 | -0.51697 | -6.1493 | greater | 1 |
| MEM85 | -0.5271 | -6.22317 | greater | 1 |
| MEM86 | -0.53228 | -6.49504 | greater | 1 |
| MEM87 | -0.56731 | -6.57326 | greater | 1 |
| MEM88 | -0.57993 | -7.11243 | greater | 1 |
| MEM89 | -0.5975 | -6.87851 | greater | 1 |
| MEM90 | -0.60511 | -7.47256 | greater | 1 |
| MEM91 | -0.62705 | -7.31809 | greater | 1 |
| MEM92 | -0.63905 | -7.26116 | greater | 1 |
| MEM93 | -0.64354 | -7.68563 | greater | 1 |
| MEM94 | -0.64679 | -7.77409 | greater | 1 |
| MEM95 | -0.67512 | -8.18407 | greater | 1 |
| MEM96 | -0.6845 | -8.31769 | greater | 1 |
| MEM97 | -0.70478 | -8.7834 | greater | 1 |
| MEM98 | -0.71278 | -8.79411 | greater | 1 |
| MEM99 | -0.75825 | -9.08226 | greater | 1 |
| MEM100 | -0.79135 | -9.86217 | greater | 1 |
| MEM101 | -0.79719 | -10.3268 | greater | 1 |
| MEM102 | -0.80591 | -10.0173 | greater | 1 |
| MEM103 | -0.81159 | -10.241 | greater | 1 |
| MEM104 | -0.82422 | -10.1243 | greater | 1 |
| MEM105 | -0.83742 | -11.0436 | greater | 1 |
| MEM106 | -0.85271 | -10.4442 | greater | 1 |
| MEM107 | -0.86892 | -10.682 | greater | 1 |
| MEM108 | -0.8893 | -10.9817 | greater | 1 |
| MEM109 | -0.89454 | -11.9991 | greater | 1 |
| MEM110 | -0.90352 | -10.8562 | greater | 1 |
| MEM111 | -0.90595 | -10.9311 | greater | 1 |
| MEM112 | -0.93121 | -12.2321 | greater | 1 |
| MEM113 | -0.94705 | -12.2964 | greater | 1 |
| MEM114 | -0.94784 | -11.6459 | greater | 1 |
| MEM115 | -0.9599 | -12.2328 | greater | 1 |
| MEM116 | -0.99776 | -12.4405 | greater | 1 |
| MEM117 | -1.01043 | -13.0083 | greater | 1 |

**Table S2** Spearman's correlation between the abundances and selected biotic and abiotic factors. Significant correlations (*P* < 0.05) are indicated as bold fonts.

|  |  | **Bacterial NMDS1** | **Fungal NMDS1** | **Protistan NMDS1** | **MAT (°C)** | **MAP (mm)** | **TC (%)** | **TN (%)** | **pH** |
| --- | --- | --- | --- | --- | --- | --- | --- | --- | --- |
| **Phyllosphere** | **Beta-lactamase**  **resistance** | **0.613** | **-0.331** | **0.281** | -0.030 | -0.157 | -0.117 | -0.039 | 0.092 |
|  |  | ***P <* 0.001** | ***P < 0.001*** | ***P =* 0.004** | *P* = 0.768 | *P* = 0.115 | *P* = 0.242 | *P* = 0.694 | *P* = 0.358 |
|  | **Multidrug resistance** | **0.505** | **-0.230** | 0.174 | -0.129 | -0.053 | -0.048 | 0.002 | 0.004 |
|  |  | ***P <* 0.001** | ***P =* 0.019** | *P =* 0.081 | *P* = 0.197 | *P* = 0.597 | *P* = 0.632 | *P* = 0.983 | *P* = 0.971 |
| **Soil** | **Multidrug resistance** | **0.121** | 0.093 | 0.007 | **0.201** | 0.101 | **0.205** | **0.221** | 0.053 |
|  |  | ***P* = 0.049** | *P* = 0.129 | *P* = 0.907 | ***P* = 0.001** | *P* = 0.099 | ***P* < 0.001** | ***P* < 0.001** | *P* = 0.387 |

**
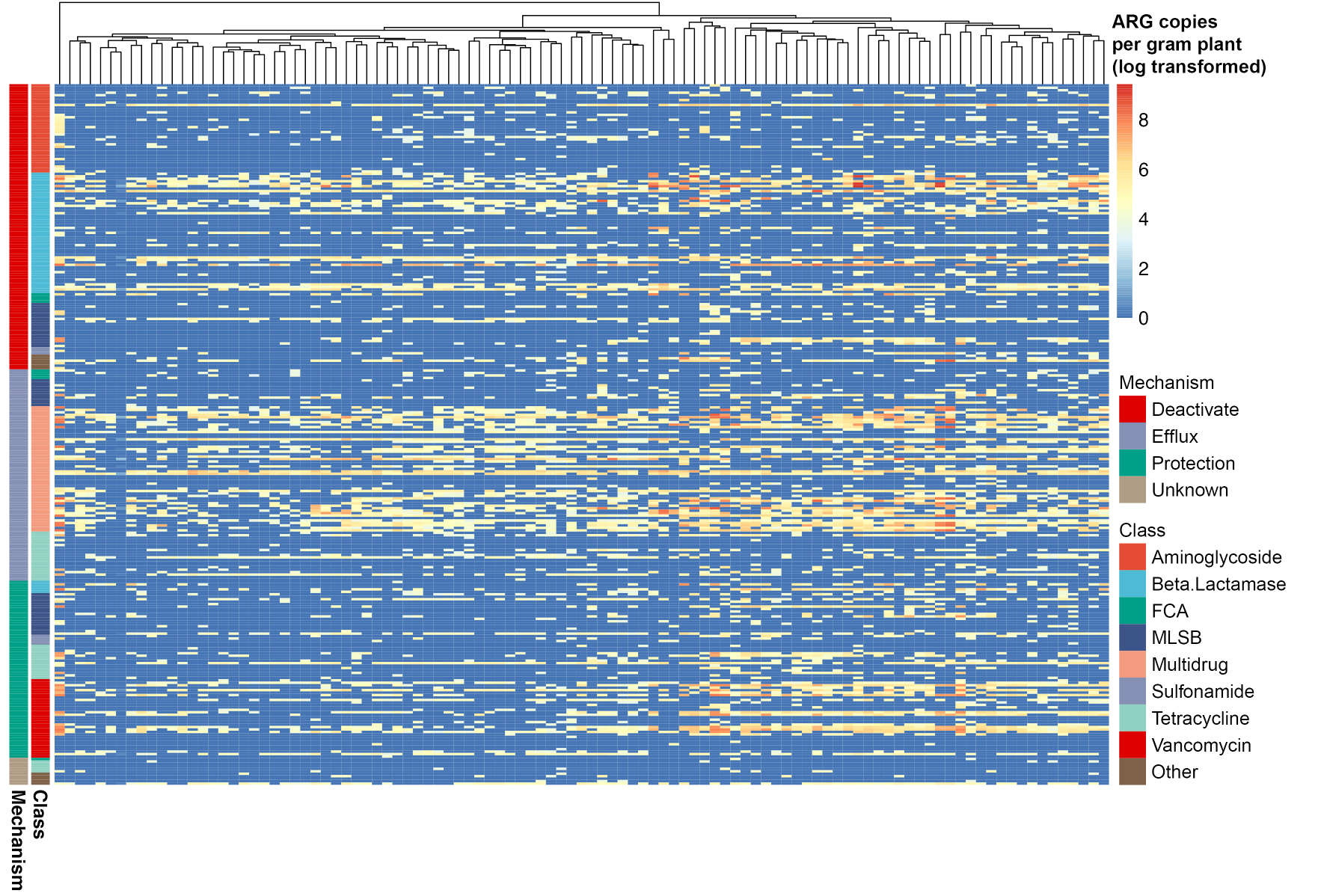
**

**Fig. S1** Heatmap showing the abundance of the ARGs detected in the phyllosphere samples.


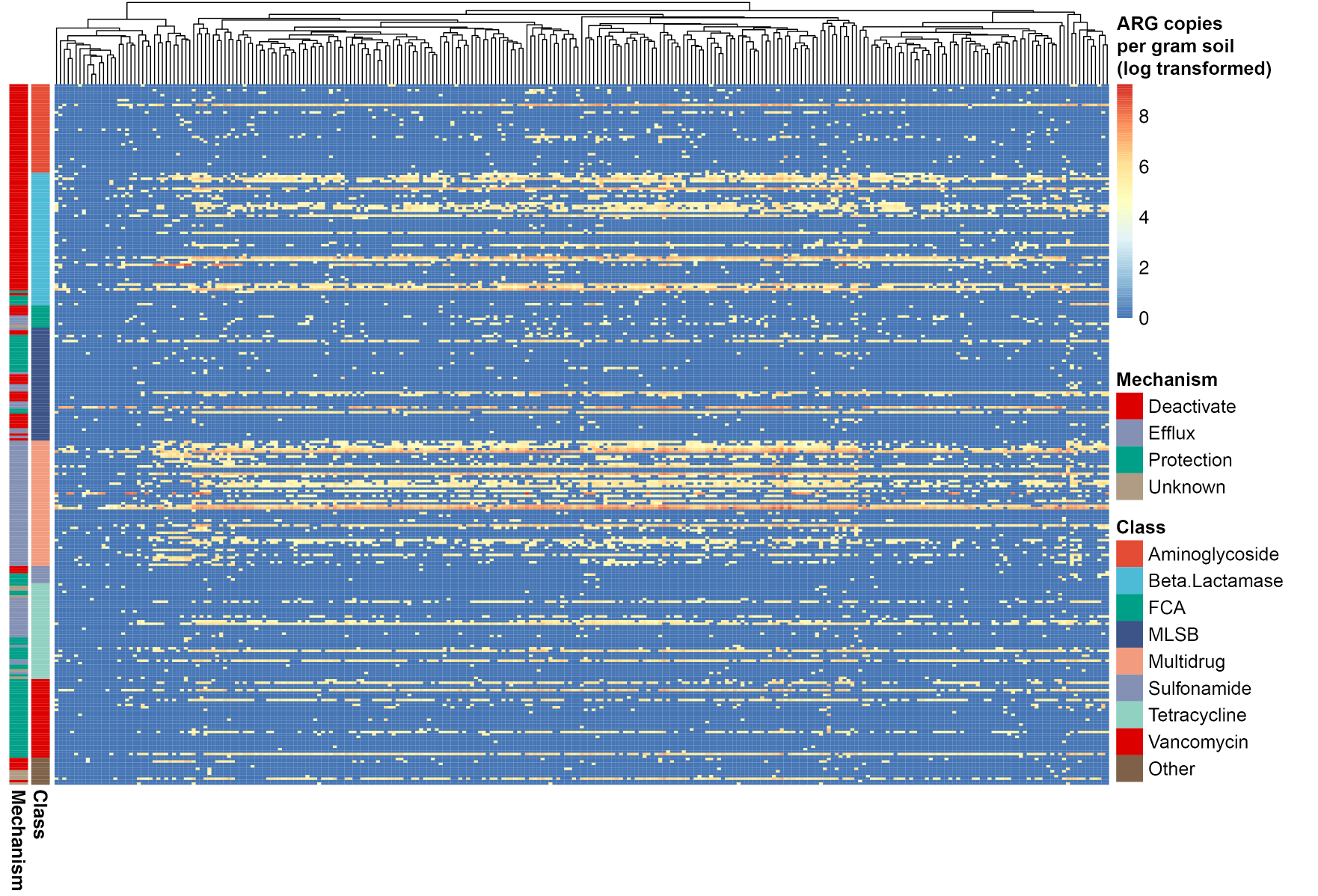


**Fig. S2** Heatmap showing the abundance of the ARGs detected in the soil samples.


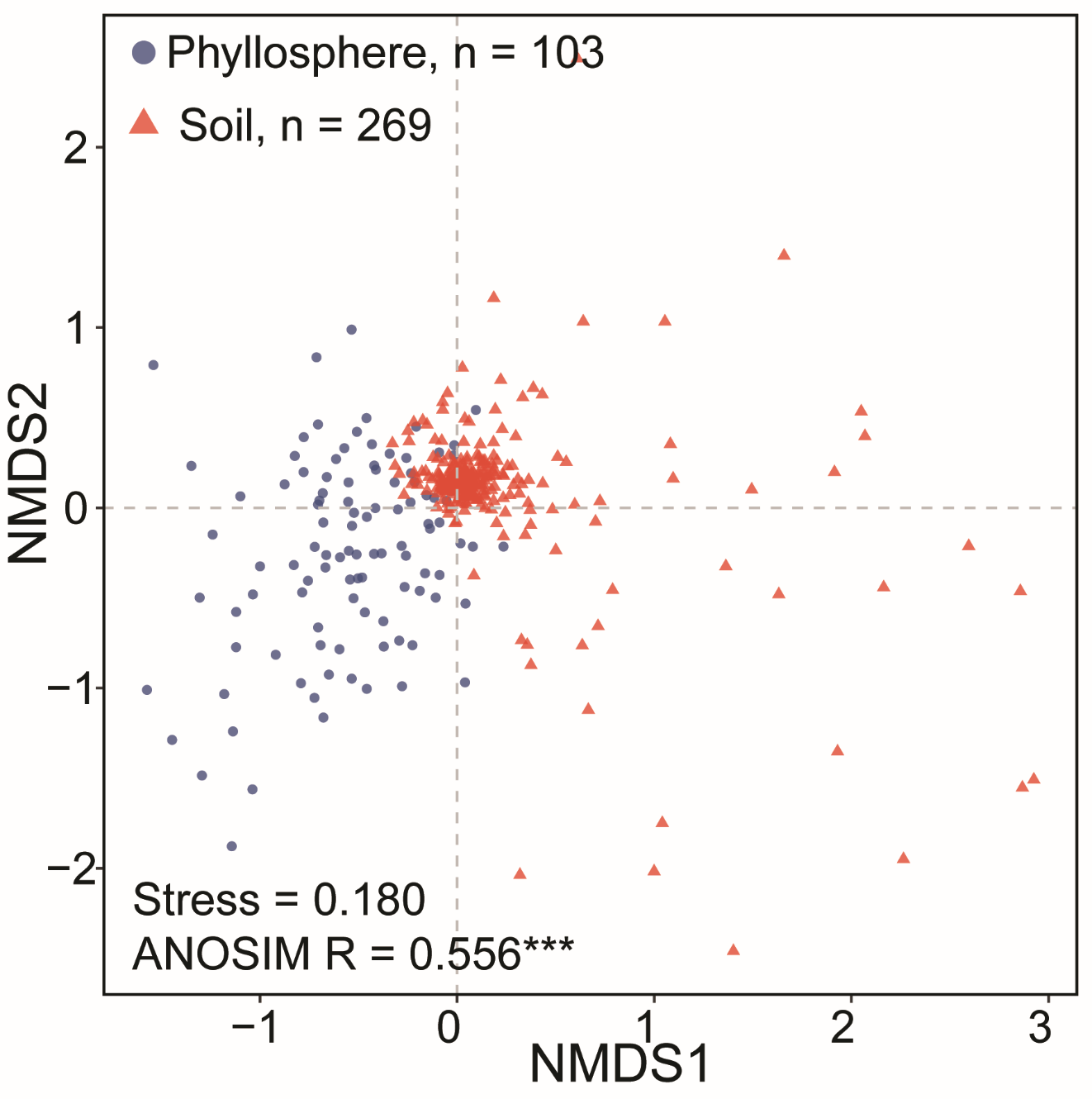


**Fig. S3** Nonmetric multidimensional scaling ordinations showing the ARG profiles in phyllosphere and soils based on the Bray-Curtis distance and ARG abundances (copies per gram sample).

**
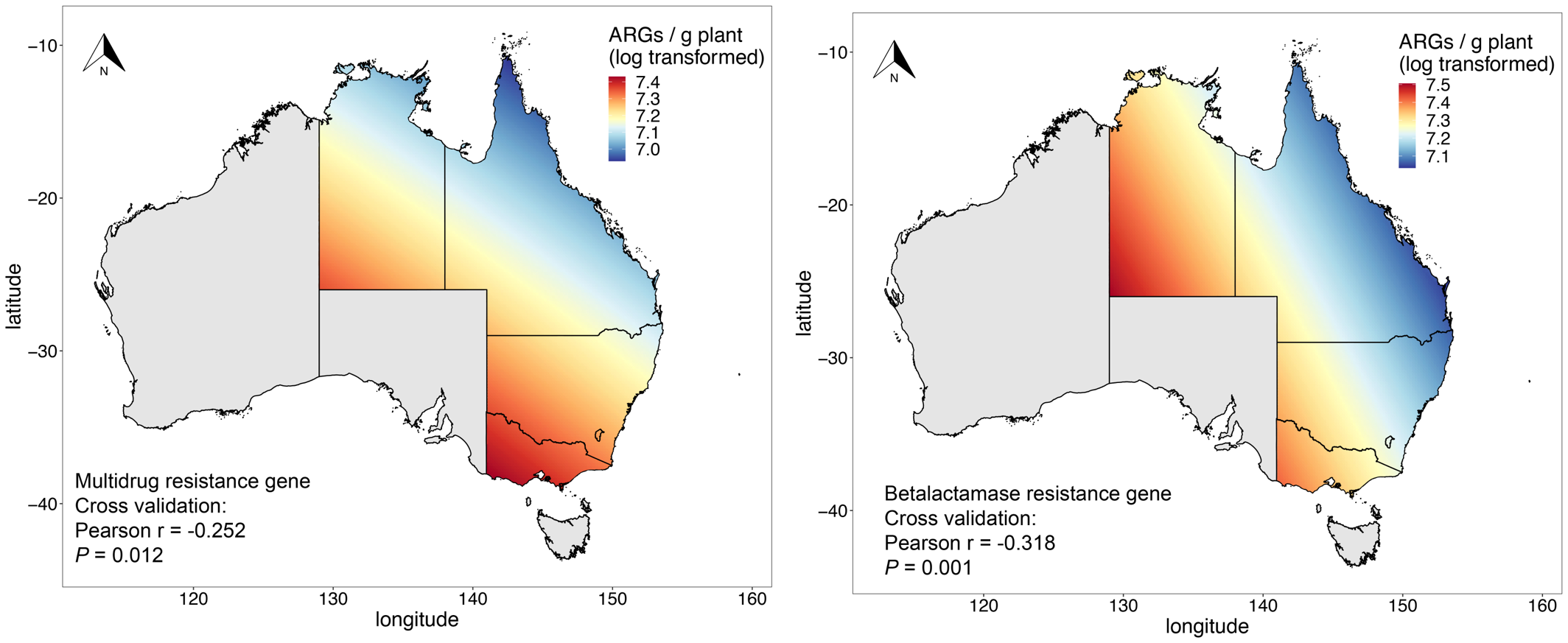
**

**Fig. S4** Large-scale atlas of the abundances of the dominant ARG classes in phyllosphere across eastern and northern Australia.

**
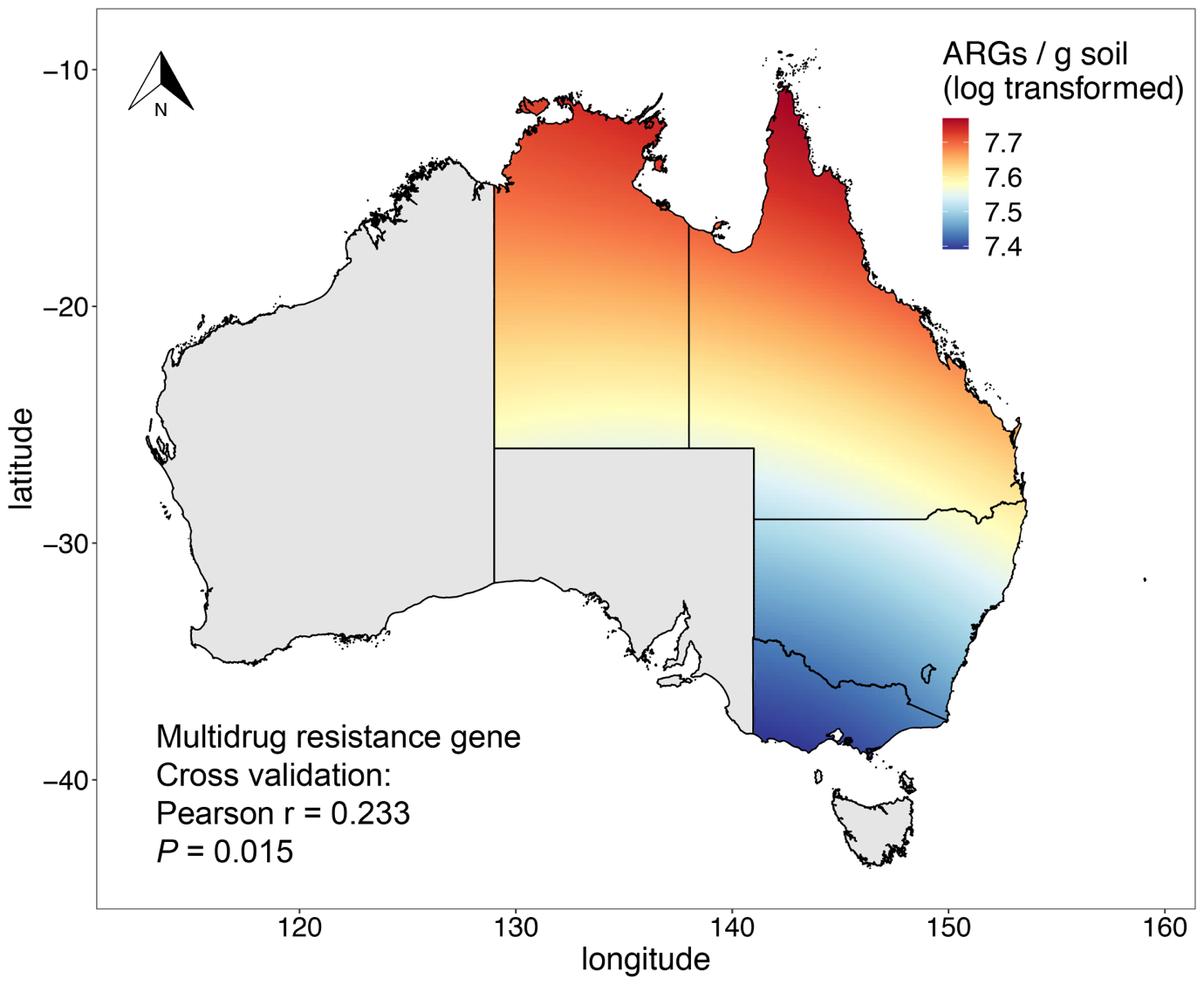
**

**Fig. S5** Large-scale atlas of the abundance of the dominant ARG class in soils across eastern and northern Australia.


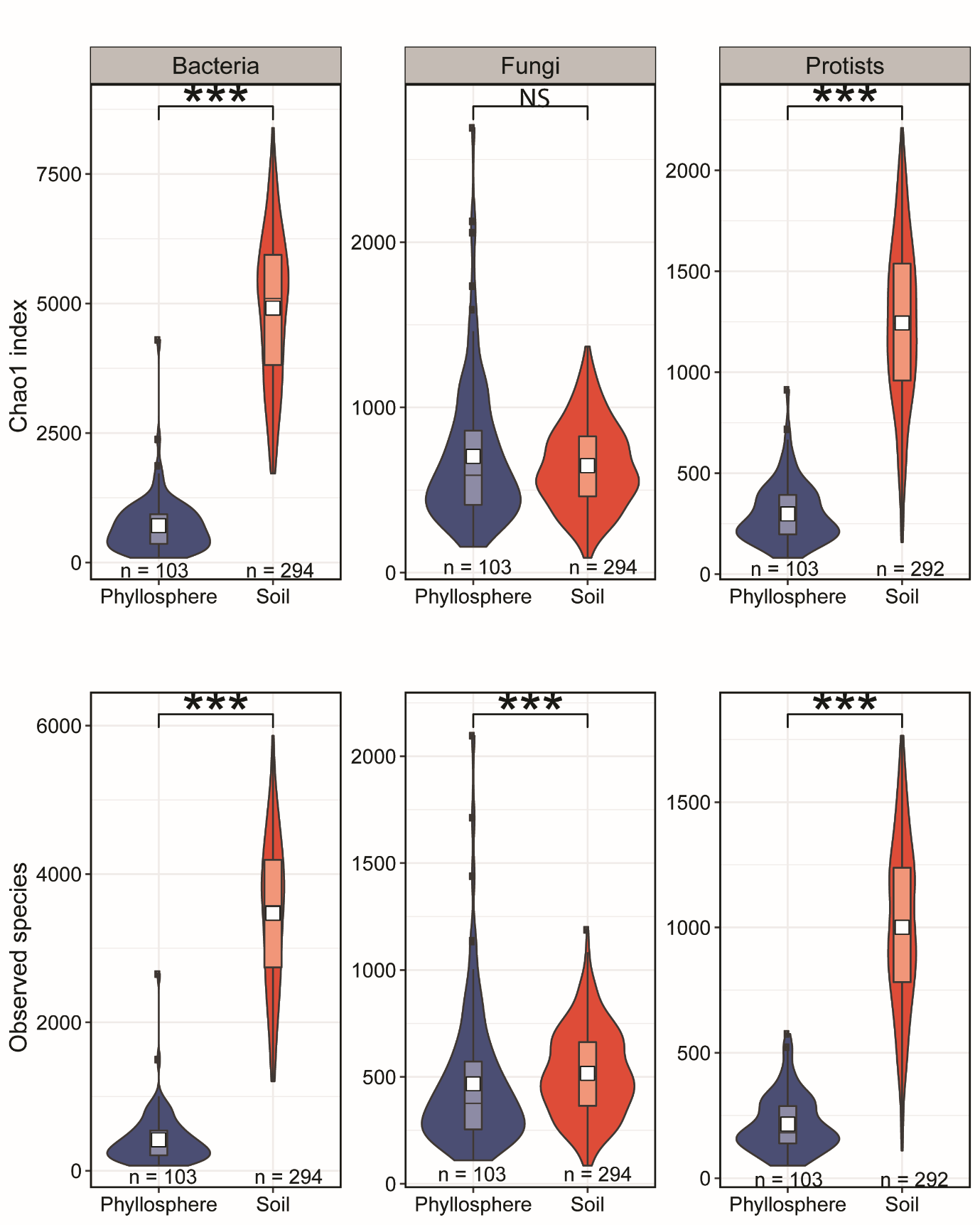


**Fig. S6** Violin plots and boxplots showing the alpha-diversity index of bacterial, fungal, and protistan communities in the phyllosphere and soils as revealed by the numbers of observed species. *** indicates Wilcoxon rank0sum test *P* < 0.001. White squares inside the box plots indicate the mean values.

**
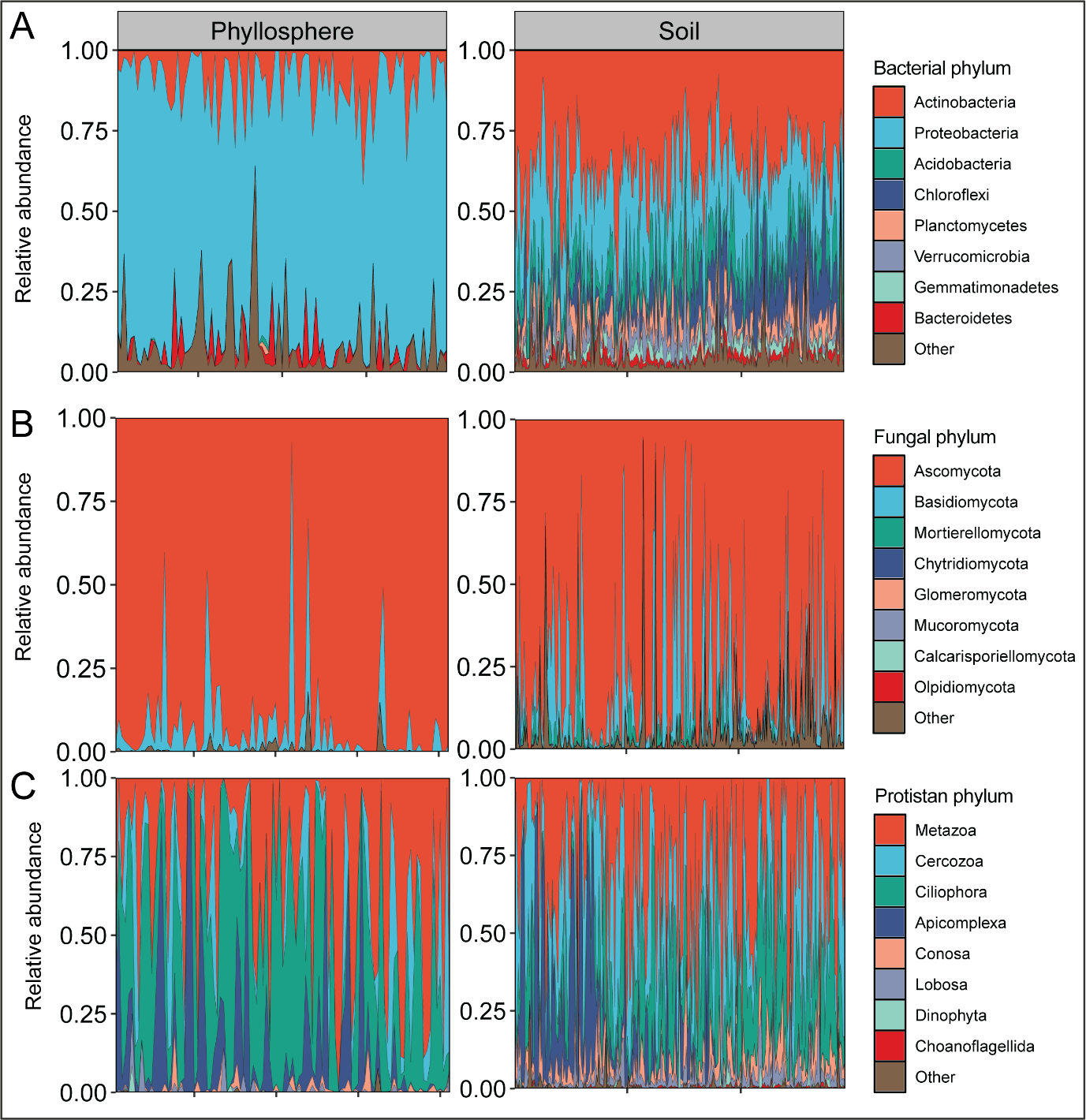
**

**Fig. S7** Area charts showing taxonomic compositions of the bacterial (A), fungal (B), and protistan (C) communities at the phylum level. Samples were ordered by ascending in the latitude of the sampling locations.


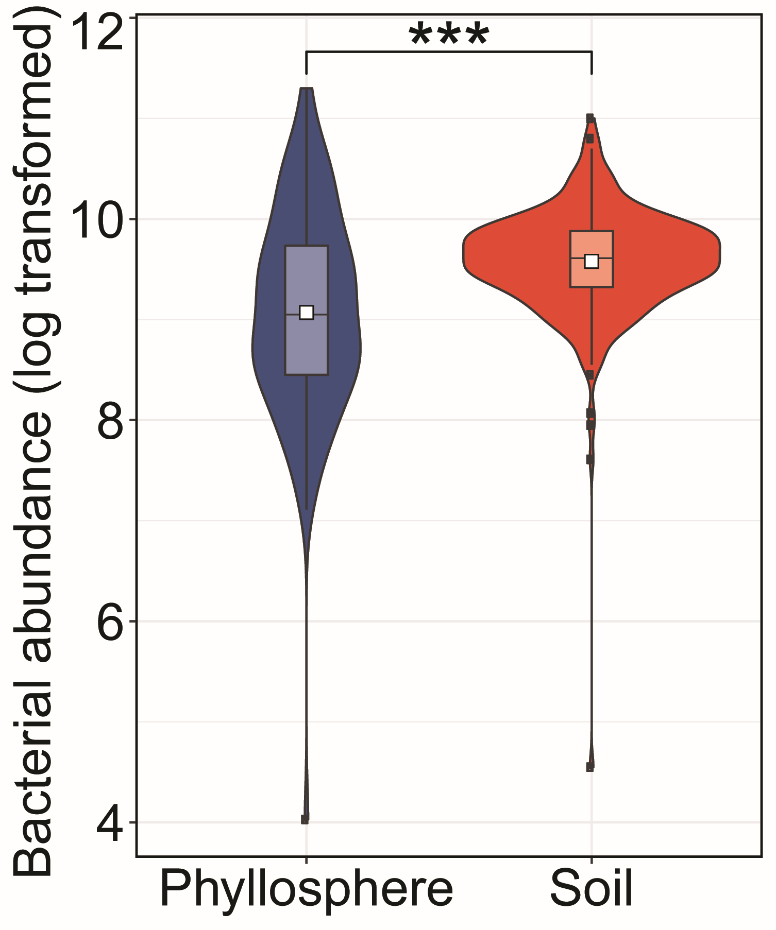


**Fig. S8** Violin plots and boxplots showing bacterial abundances in the phyllosphere and soils as revealed by copy numbers of 16S rRNA gene. *** indicates Wilcoxon rank0sum test *P* < 0.001. White squares inside the box plots indicate the mean values

**
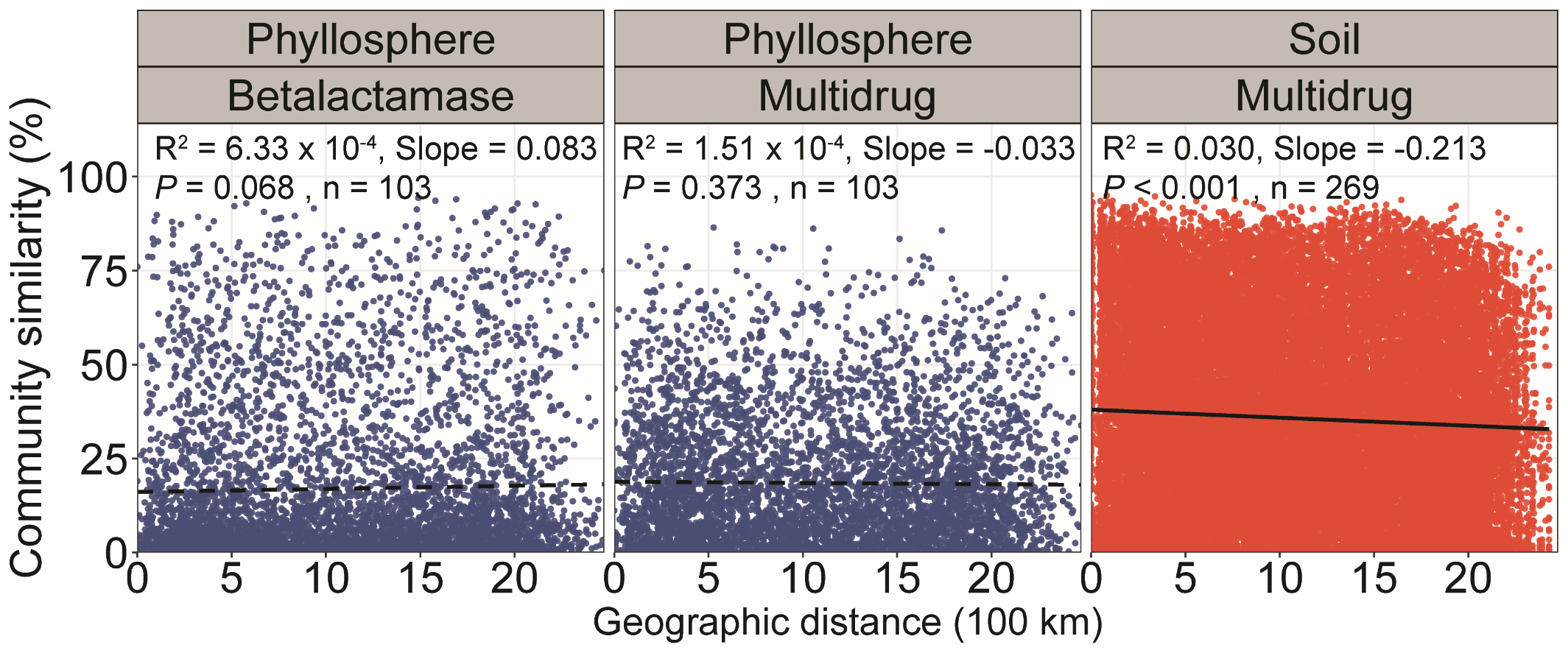
**

**Fig. S9** Distance-decay relationships for the dominant ARG class(es) in phyllosphere and soils. *** indicates ordinary least square regression *P* < 0.001. Significant correlation (*P* < 0.05) and not significant correlations (*P* > 0.05) are shown in solid line and dashed lines, respectively.

**
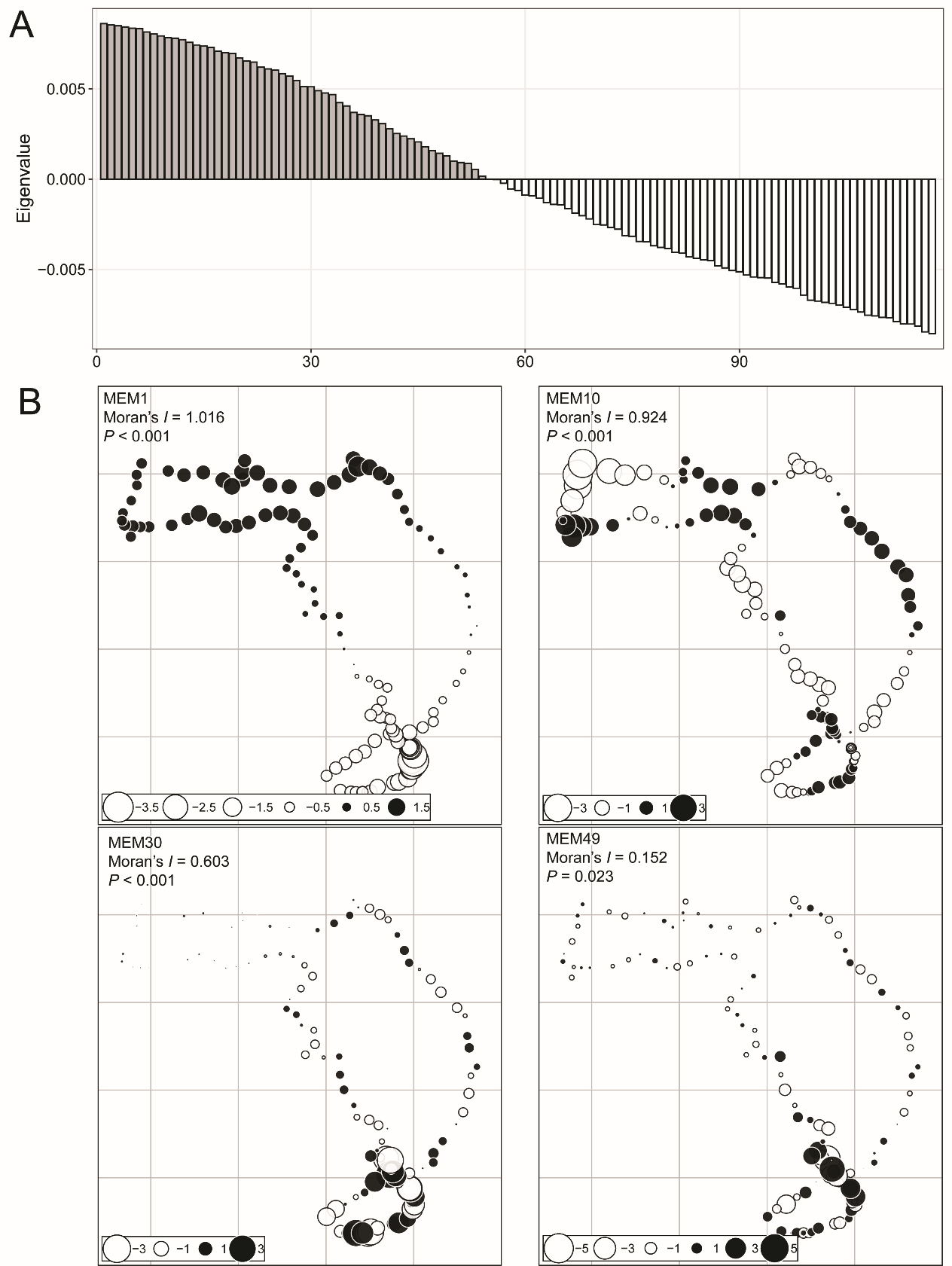
**

**Fig. S10** (A) Eigenvalues of the spatial weighting matrix of the Moran’s eigenvector maps (MEMs). (B) Examples of MEMs describing spatial patterns. MEM1 and MEM10 describe large scale spatial autocorrelation. MEM30 and MEM49 represent specific patterns in corners of the sampling area at the inter-mediate scale.

**
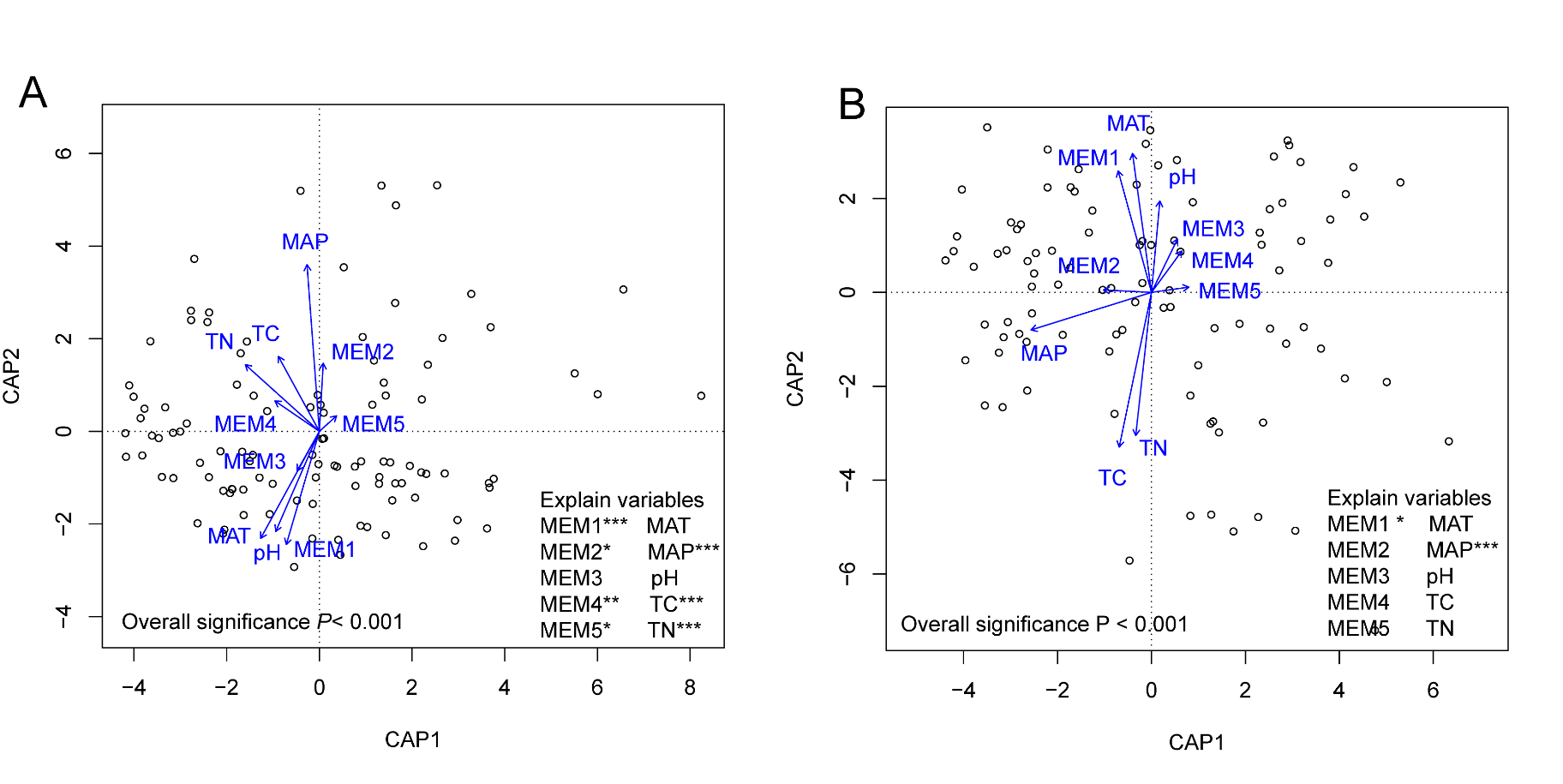
**

**Fig. S11** Distance-based redundancy analysis results showing the impacts of changes in spatial patterns, edaphic and climatic factors in the variances in soil (A) and phyllosphere (B) resistome profiles. * *P* < 0.05, ** *P* < 0.01, *** *P* < 0.001. MEM1 -5: Top five largest eigenvalues of the spatial weighting matrix of the Moran’s eigenvector maps. MAT: mean annual temperature (°C). MAP: mean annual precipitation (mm). TC: Soil total carbon (%). TN: soil total nitrogen (%).

**
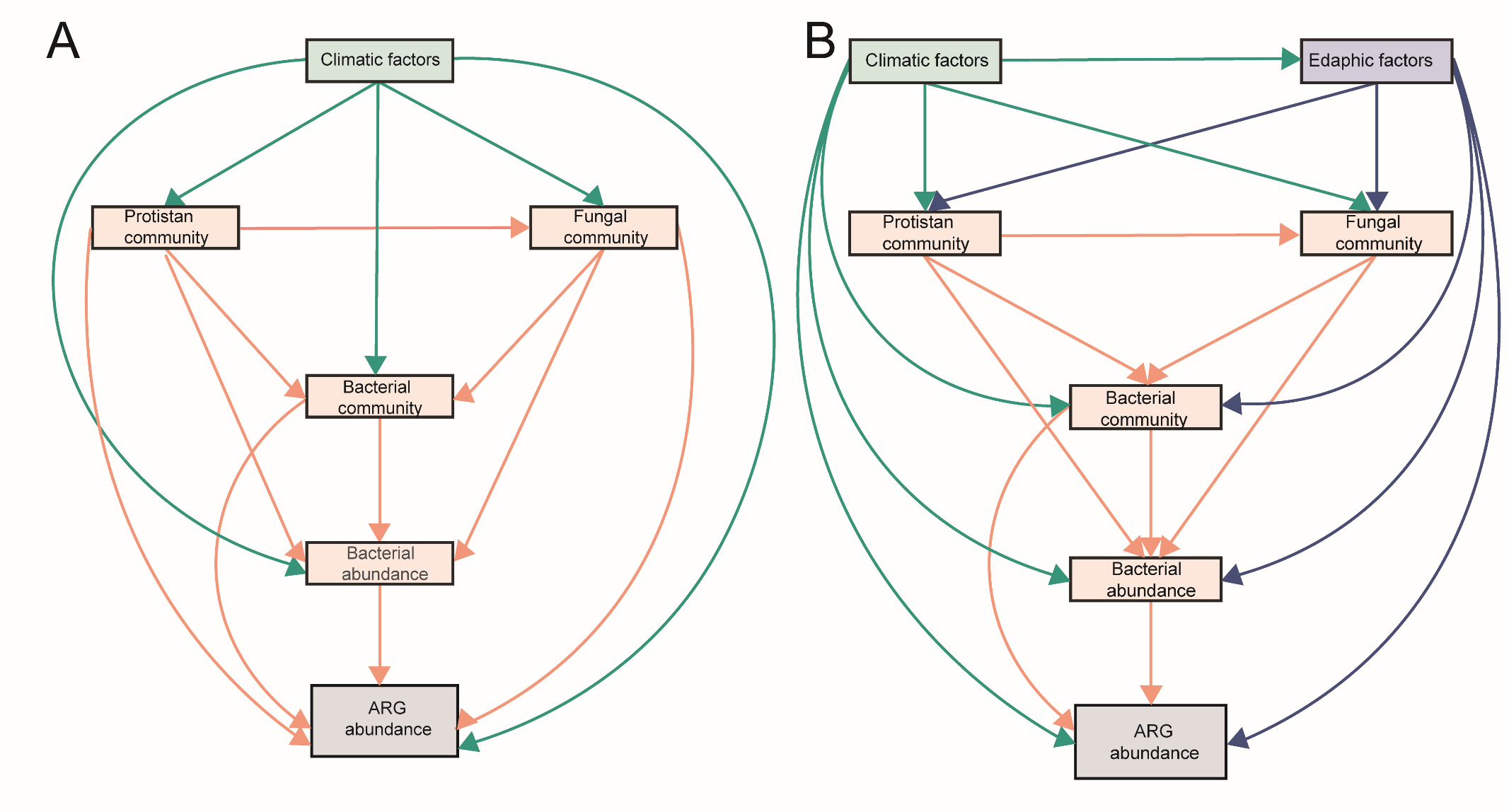
**

**Fig. S12** *A priori* models for the structure equation models of variation in ARG abundances in phyllosphere (A) and soils (B) based on the hypothesized causal relationships between multiple factors and ARG abundances.
